# Effective Activation of Strong C−Cl Bonds for Highly Selective Photosynthesis of Bibenzyl via Homo‐Coupling

**DOI:** 10.1002/anie.202307907

**Published:** 2023-09-18

**Authors:** Qingning Yang, Xiyi Li, Lu Chen, Xiaoyu Han, Feng Ryan Wang, Junwang Tang

**Affiliations:** ^1^ Department of Chemical Engineering University College London Torrington Place London WC1E 7JE UK; ^2^ Department of Chemistry University of Manchester Manchester M13 9PL UK; ^3^ Industrial Catalysis Centre, Department of Chemical Engineering Tsinghua University Beijing 100084 China

**Keywords:** Bibenzyl Synthesis, C−Cl Bond Activation, Cu Species, Heterogeneous Photocatalysis

## Abstract

Carbon‐carbon (C−C) coupling of organic halides has been successfully achieved in homogeneous catalysis, while the limitation, e.g., the dependence on rare noble metals, complexity of the metal‐ligand catalylst and the poor catalyst stability and recyclability, needs to be tackled for a green process. The past few years have witnessed heterogeneous photocatalysis as a green and novel method for organic synthesis processes. However, the study on C−C coupling of chloride substrates is rare due to the extremely high bond energy of C−Cl bond (327 kJ mol^−1^). Here, we report a robust heterogeneous photocatalyst (Cu/ZnO) to drive the homo‐coupling of benzyl chloride with high efficiency, which achieves an unprecedented high selectivity of bibenzyl (93 %) and yield rate of 92 % at room temperature. Moreover, this photocatalytic process has been validated for C−C coupling of 10 benzylic chlorides all with high yields. In addition, the excellent stability has been observed for 8 cycles of reactions. With detailed characterization and DFT calculation, the high selectivity is attributed to the enhanced adsorption of reactants, stabilization of intermediates (benzyl radicals) for the selective coupling by the Cu loading and the moderate oxidation ability of the ZnO support, besides the promoted charge separation and transfer by Cu species.

## Graphical abstract

Cu loaded ZnO nanoparticles exhibit robust performance in photocatalytic C−C coupling of inert benzyl chloride. Compared to previously reported Cu/TiO_2_, the selectivity towards bibenzyl increases from 57 to 93 %. Cu loading enhances the reactant adsorption, C−Cl bond activation and charge separation/transfer. The moderate oxidation of 2‐propanol by ZnO is a key factor in alleviating the generation of byproduct toluene.

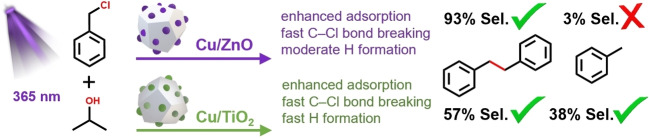

## Introduction

Carbon‐carbon (C−C) coupling is one of the most fundamental reactions in modern organic synthesis.[^1^, ^2^] Dihydrostilbenoids, as natural phenols, are often synthesized using the key chemical of bibenzyl. Due to dihydrostilbenoids’ wide applications[^3^, ^4^, ^5^, ^6^] in the pharmaceutical and chemical industry and their scarce existence in nature,[^7^, ^8^, ^9^, ^10^] the synthesis of bibenzyl compounds has gained significant attention among various coupling reactions in the past decades.[^10^, ^11^, ^12^] However, harsh reaction conditions (e.g., high temperature) and complicated and high‐cost catalysts (e.g., noble metals and designed metal‐complex) are often needed to achieve high yield and selectivity of bibenzyls.[^10^, ^13^, ^14^, ^15^]

Heterogeneous photocatalysis, as a growing field in organic synthesis, has shown great potential for various C−C coupling reactions, where the reactions can proceed under mild conditions with green energy sources (e.g., solar energy). Furthermore, compared to homogeneous photocatalysis, heterogeneous photocatalysis also exhibits the superiority of outstanding recyclability, high stability and easy separation of the products from solvents and catalysts. Recently, a series of heterogeneous photocatalysts have been reported for the C−C homo‐coupling reaction of different alkyl halides.

For example, colloidal CsPbBr_3_ perovskite was reported as a photosensitizer for the homo‐coupling of benzyl bromides (7 examples, yield 42–83 %) with TON as high as 17500, but this perovskite could only be used twice, and aprotic solvent and organic scavenger with a prolonged reaction time were required (20–48 h).^[16]^ Modified AgGaO_2_ with shape engineering was synthesized, and the flat AgGaO_2_ with electron‐rich (001) facets exhibited 20 times higher activity compared to (012) facets stretched AgGaO_2_ in dehalogenative homo‐coupling of benzyl bromides (5 examples, yield 75–83 %).^[17]^ The hole‐rich stretched AgGaO_2_, on the other hand, showed better photocatalytic performance in the oxidative homo‐coupling of aniline, extending shape engineering in tuning selectivity for coupling reactions. Surface hydroxylation modified graphitic carbon nitride (gCN‐OH) was reported to efficiently activate water and use it as hydrogen donors under mild conditions. It could be applied to a series of coupling reactions, including homo‐coupling of benzyl bromides (11 examples, yield 80–91 %).^[18]^ All these indicate a clean route for organic synthesis using renewable energy.

Alkyl halides are important reactants in constructing C−C bond for fine chemicals, pharmaceutically active compounds and agricultural chemicals.^[19]^ Compared with alkyl chloride, alkyl bromides have been used and reported more intensively[^16^, ^17^, ^18^, ^20^, ^21^, ^22^, ^23^, ^24^] because C−Br bond has a much lower energy (285 kJ mol^−1^) than that of C−Cl (327 kJ mol^−1^),^[25]^ so the former is more easily activated and the product selectivity is also well controlled. However, alkyl chlorides have superior properties than alkyl bromides,^[26]^ such as low‐cost and abundance and reduced toxicity. Due to its inert nature, alkyl chlorides have been considered as sluggish reactants in organic synthesis.[^19^, ^27^, ^28^] It is really challenging to achieve high yield and selectivity using alkyl chloride for the C−C homocoupling reaction.

Herein, after screening a series of noble metals and transition metals, copper was selected as the best co‐catalyst to decorate the robust semiconductor ZnO forming the efficient photocatalyst to drive the homo‐coupling of benzyl chlorides at room temperature. With the manipulation of electron donors, hydrogen donors and water content, the optimized sample 1.5 % Cu/ZnO achieves a 99 % conversion with a 93 % selectivity to desired product bibenzyl. Such excellent performance is also stable for up to 8 cycles. Furthermore, the university of this low‐cost system has been confirmed by 11 various chloride derivatives with a good or very high yield (60–96 %). The high selectivity is attributed to the synergetic effect between Cu and ZnO, in which benzyl radicals are stabilized on Cu for further coupling and protons are adsorbed on the ZnO support. In addition, the high yield rate is also beneficial from the promoted charge transfer between ZnO and Cu.

## Results and Discussion

### Catalytic Performance

**Figure 1:**
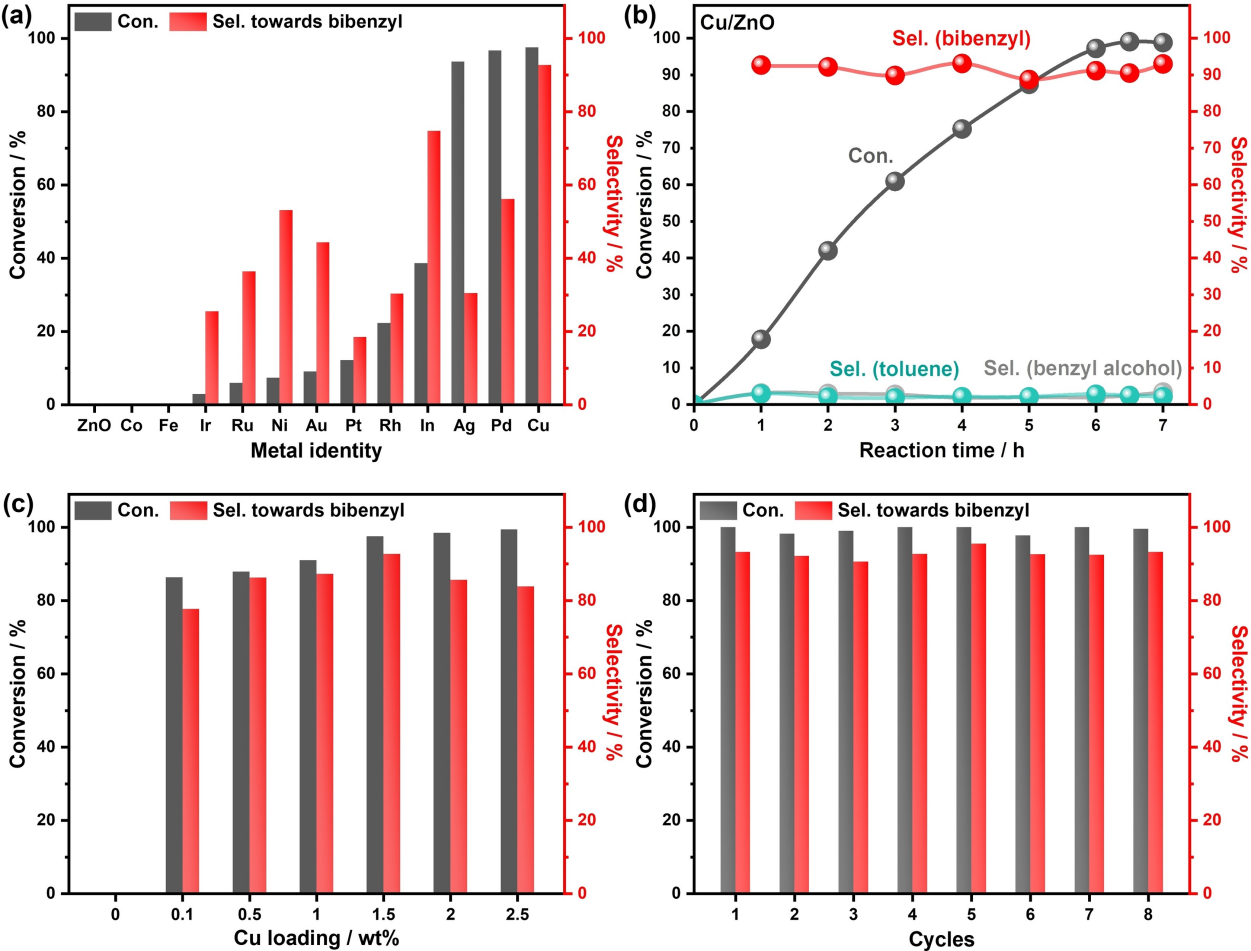
Benzyl chloride coupling over different photocatalysts. (a) Photocatalytic benzyl chloride coupling using a series of selected metals decorated ZnO, 1.5 wt % M/ZnO (M=Cu, Pd, Ag, In, Rh, Au, Ni, Pt, Ru, Ir, Fe, Co). (b) Temporal study of photocatalytic benzyl chloride conversion over 1.5 wt % Cu/ ZnO. (c) Effect of Cu loading amount on the photocatalytic coupling of benzyl chloride. (d) Stability test of 1.5 wt % Cu/ZnO. Reaction conditions: 10 mM reactant, 10 mg photocatalyst in 5 ml solvent (2‐propanol: water=1 : 1) under 365 nm LED irradiation for 6 h in Ar at room temperature.

The ZnO powders were prepared by a precipitation method.^[29]^ The metal‐loaded ZnO samples were prepared by either photo‐deposition or impregnation methods (Detailed experimental procedures can be found in the Supporting Information and the reactor setup is shown in Figure S1). From the X‐ray diffraction (XRD) results (Figure S2), ZnO NPs with excellent crystallinity were successfully synthesized, which indexed to ZnO (JCPDS no: 043‐0002). Various metals were then loaded on this ZnO support with a similar amount of 1.5 wt %. No extra peaks for the other metal species can be observed in the spectra, probably due to either low concentration and/or high dispersion on the support of ZnO.^[30]^ Then, their photocatalytic activity toward homo‐coupling of benzyl chloride was evaluated, as shown in Figure 1a. After 6 h light irradiation (λ=365 nm), ZnO exhibits negligible conversion of benzyl chloride, likely owing to the high recombination rate of photoinduced electrons and holes. Among the 12 metal co‐catalysts tested, most of them display low conversion of benzyl chloride (<22 %) and low selectivity (<50 %) towards bibenzyl (detailed activity is summarized in Table S1, entries 1–13). Two possible reasons are behind this result. Some metals (i.e., Ir, Rh, Ni, Pt) show high performance for the dehydrogenation of alcohols,[^31^, ^32^] so there is a great possibility that the adsorbed benzyl radical would undergo hydrogenation and generate toluene as a byproduct. Some metals, such as Fe, Co and Ru, exhibit strong interactions with halides,^[33]^ which could lead to poisoning effects of co‐catalysts, hence the low activity. Surprisingly, Indium (In) displays relatively high selectivity towards bibenzyl (75 %), but the conversion of benzyl chloride is only 38 %. This is likely due to lower charge separation/transfer efficiency in the photocatalytic process. Ag, Pd and Cu show excellent conversion of over 90 % of benzyl chloride. In particular, Cu shows both a very high conversion of 99 % and selectivity of 93 % to bibenzyl (reaction equations are shown in Equation S1). As for Pd and Ag, the dehalogenation product toluene is the main byproduct with up to 44 % and 68 % selectivity, respectively, and a small amount (3 % yield) of benzyl alcohol, which is another byproduct from hydrolysis, is observed in every experiment (Gas chromatography (GC) spectra of products for 3 photocatalysts are shown in Figure S3). The temporal study of benzyl chloride homo‐coupling over 1.5 % M/ZnO (M=Cu, Pd, Ag) was then conducted. For Cu decorated ZnO (Figure 1b), the conversion of benzyl chloride exhibits a linear increase and the selectivity towards bibenzyl well remains above 90 % throughout the reaction time while only small amounts of toluene and benzyl alcohol are detected. For Pd species (Figure S4), the temporal study result shows that the selectivity to bibenzyl and toluene remain constant at around 55 % and 43 % during the reaction time, respectively. The adsorption energy of hydrogen atoms on Pd is ≈−0.43 eV,^[31]^ which is much negative compared to on Cu (≈0.05 eV), so the low selectivity to bibenzyl may be attributed to the excessive amount of surface‐adsorbed protons, which favours the conversion of the intermediate (benzyl radical) to the dehalogenation product (toluene). In addition, from the temporal study result of Ag/ZnO (Figure S5), the selectivity to bibenzyl gradually drops from 52 % to 31 % after 6 h irradiation while the selectivity towards toluene increases accordingly (from 47 % to 68 %). The reason behind the poor selectivity of Ag co‐catalyst is likely due to the poison by Cl^−^ ions that gradually convert silver species into AgCl, which has a more decisive influence on the coupling process (bibenzyl production) than on the hydrogenation process (toluene production). In order to prove this hypothesis, a higher silver concentration (5 wt %) decorated ZnO was therefore prepared. The XRD result (Figure S6) of 5 % Ag/ZnO sample after reaction shows peaks corresponding to (111), (200) and (222) lattice planes of AgCl, indicating the poison of Ag NPs by Cl^−^ ions.

Based on the screening results, Cu was then selected as the best co‐catalyst to further improve bibenzyl production. The loading amount effect of Cu on the catalytic performance was then investigated (detailed activity is summarized in Table S1, entries 14–18), as shown in Figure 1c. It should be noted that even a trace amount (low to 0.1 wt %) of Cu loading can increase the selectivity to the desired bibenzyl from 0 % to 78 %, together with more than 80 % conversion. Further increasing the loading amount of Cu can improve both conversion and selectivity accordingly, and the highest selectivity (93 %) towards bibenzyl can be achieved at 1.5 wt % Cu loading with a conversion of 100 % after 6 h reaction. Additionally, excessive Cu loading cannot further improve the conversion of benzyl chloride, and the selectivity to bibenzyl shows a gradual decrease. This is likely owing to the increase of CuO_x_ particle size with excessive Cu covering on the surface.^[34]^ Inductively coupled plasma atomic optical emission spectroscopy (ICP‐OES) was used to determine the actual amount of Cu loaded for the 1.5 wt % Cu/ZnO sample, and the loading amount of Cu is measured to be 1.42 wt %, very close to the nominal value. Therefore, 1.5 wt % Cu/ZnO is selected as the best candidate for the subsequent investigation (denoted Cu/ZnO thereafter).

The effect of solvents was then investigated, as shown in Figure S7. After light irradiation for 3 h, the systems using tetrahydrofuran (THF), acetonitrile (MeCN) and dimethylformamide (DMF) show trace or even no yield of bibenzyl. As all three aprotic solvents are not suitable electron donors as reported due to their higher oxidation potentials,[^35^, ^36^] it is unfavourable to inject electrons into the photocatalyst to facilitate the reduction half‐reaction for the dehalogenative process. Then, alcohols as widely used electron donors in photocatalysis^[37]^ were applied, including methanol, ethanol, 1‐propanol and 2‐propanol. Expectedly, the yield of bibenzyl can be observed after the use of alcohol solvents and the highest selectivity of 63 % is achieved when 2‐propanol is used as the solvent. It is interesting to find that the yield of undesired byproduct toluene decreases with the extension of the carbon chain of the alcohols used (methanol>ethanol>1‐propanol).^[38]^ Longer carbon chains may result in stronger steric effects, offering less interaction opportunities for the alcohol to directly donate the proton to the key intermediate (benzyl radical) to form toluene. Thus, it is reasonable to see 2‐propanol as secondary alcohol shows the least selectivity (only 3 %) to toluene as its hydroxyl group at the middle carbon atom rather than the terminal position, which is less accessible.

Additionally, different water contents in the solvent (0 %, 30 %, 50 %, 70 %) were investigated by taking into account the possible influence of solubility (Figure S8). With the addition of water, the selectivity to bibenzyl increases. Notably, high bibenzyl selectivity of 92 % with 78 % conversion of benzyl chloride can be reached when 50 % content of water is added after 3 h reaction. This may be due to the higher solubility of HCl (the other dehalogenation product) in water, likely shifting the reaction equilibrium to form more bibenzyl.^[39]^ While too much water (e.g., 70 %) may decrease the solubility of benzyl chloride in a protic organic solvent, leading to poor dispersion and interaction between the substrate and the catalyst. In addition, In the presence of 50 % water content in the solvent, a small amount of benzyl chloride would undergo hydrolysis and react with water to form benzyl alcohol (3 % yield) and hydrochloric acid.^[40]^ The yield of benzyl alcohol increases when the water content reaches 70 %, suggesting protonic concentration is critical for hydrolysis of benzyl chloride.

Finally, the stability, efficiency and scaling‐up of bibenzyl photosynthesis via benzyl chloride over Cu/ZnO were evaluated. During the stability and durability test, the Cu/ZnO was separated and collected after each run by filtrating the reaction mixture. After rinsing with water, the catalyst was dried in a vacuum oven. The recycled Cu/ZnO photocatalyst was reused in follow‐up reactions under the same conditions. According to stability results (Figure 1d), the yield of bibenzyl well remains over 90 % with no obvious decay after 8 cycles, indicating high stability of Cu/ZnO photocatalyst for homo‐coupling of benzyl chloride. At low light intensity (27 mW cm^−2^), the Cu/ZnO photocatalyst possesses an apparent quantum efficiency (AQE) of 2 %, suggesting no convincing evidence for the radical propagation process. The low AQE value also indicates the challenge of activating C−Cl bonds (detailed calculation can be found in Supporting Information). Furthermore, In the presence of a higher benzyl chloride concentration (40 mM, Figure S9), the high conversion of benzyl chloride can be achieved by simply extending the irradiation time (40 h). Notably, the higher reactant concentration and prolonged irradiation do not compromise the selectivity towards bibenzyl, which remains consistently high at 90 %. These results suggest that this approach has the potential for practical implementation.

### Substrate Scope

**Figure 2:**
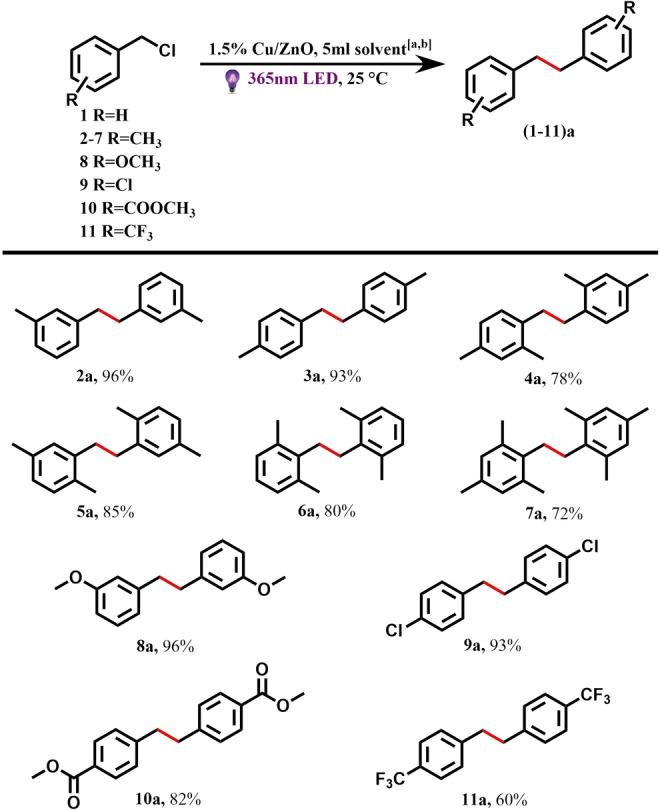
Extension to different substrates. Photocatalytic homo‐coupling of various benzylic chlorides to the corresponding bibenzyls using Cu/ZnO photocatalyst. (a) Reaction conditions: 10 mM reactant, 10 mg photocatalyst in 5 ml solvent (2‐propanol: water=1 : 1) under 365 nm LED irradiation for 6 h, Ar, room temperature. (b) Analysed by gas chromatography‐flame ionization detection (GC‐FID) and gas chromatography‐mass spectrometry (GC‐MS), and all conversions ≥99 %, the percentage indicates its yield, the equation for calculating yield is shown in Equation S2.

The reaction system was further extended to 10 alkyl chlorides with various functional groups to examine the general applicability of Cu/ZnO for photocatalytic C−C homo‐coupling reactions, as shown in Figure 2. The Cu/ZnO photocatalyst can efficiently drive the homo‐coupling of the benzylic chlorides with different methyl‐groups. For example, both 3‐methylbenzyl chloride (2) and 4‐methylbenzyl chloride (3) can be efficiently converted to the corresponding coupling products 2a and 3a, with a high yield of 96 % and 93 %, respectively. However, with increasing numbers of methyl groups on the substrates, such as (2,4‐dimethylbenzyl chloride (4), 2,5‐dimethylbenzyl chloride (5) and 2,6‐dimethylbenzyl chloride (6)), a slight decrease in the yields has been observed (78 %, 85 %, 80 % yields for 4a, 5a and 6a). Furthermore, 2,4,6‐trimethylbenzyl chloride (7) with three methyl groups shows a relatively moderate yield of 7a (72 %). The observed decrease in yield can be attributed to the steric hindrance effect.^[41]^ Then, the electronic effect of different substituents was also studied. When a strong electron‐donating group, methoxy, is added to the benzene ring, the corresponding coupling product 8a is obtained with a very high selectivity of 96 %. This is because the higher electron density of the benzene ring can facilitate the reduction reaction to release the Cl substituent, which is a rate‐determining step in this process.^[42]^ In contrast, when electron‐withdrawing groups are introduced (e.g., −Cl, −COOMe), the yields of desired products 9a and 10a show a slight decrease, but it is still as high as 82 % and 93 %. Furthermore, 4‐trifluoromethylbenzyl chloride (11) with the strongest electron‐withdrawing group −CF_3_ can still give a product yield of 60 % towards 11a. Electron‐withdrawing groups change the reactivity of a molecule by reducing the electron density on adjacent carbon atoms,^[43]^ leading to a more electron deficient carbon centre and a weaker reactivity. The observed yields of 9a, 10a and 11a (93 % for −Cl, 82 % for −COOMe and 60 % for −CF_3_) align with their respective electron‐withdrawing abilities (−Cl<−COOMe<−CF_3_). The above results show that electron‐donating groups favor this synthetic process. It also indicates that Cu/ZnO shows excellent tolerance to a wide substrate scope, and the nuclear magnetic resonance (^1^H NMR and ^13^C NMR) results are shown in Figure S10–S19.

### Characterizations

**Figure 3:**
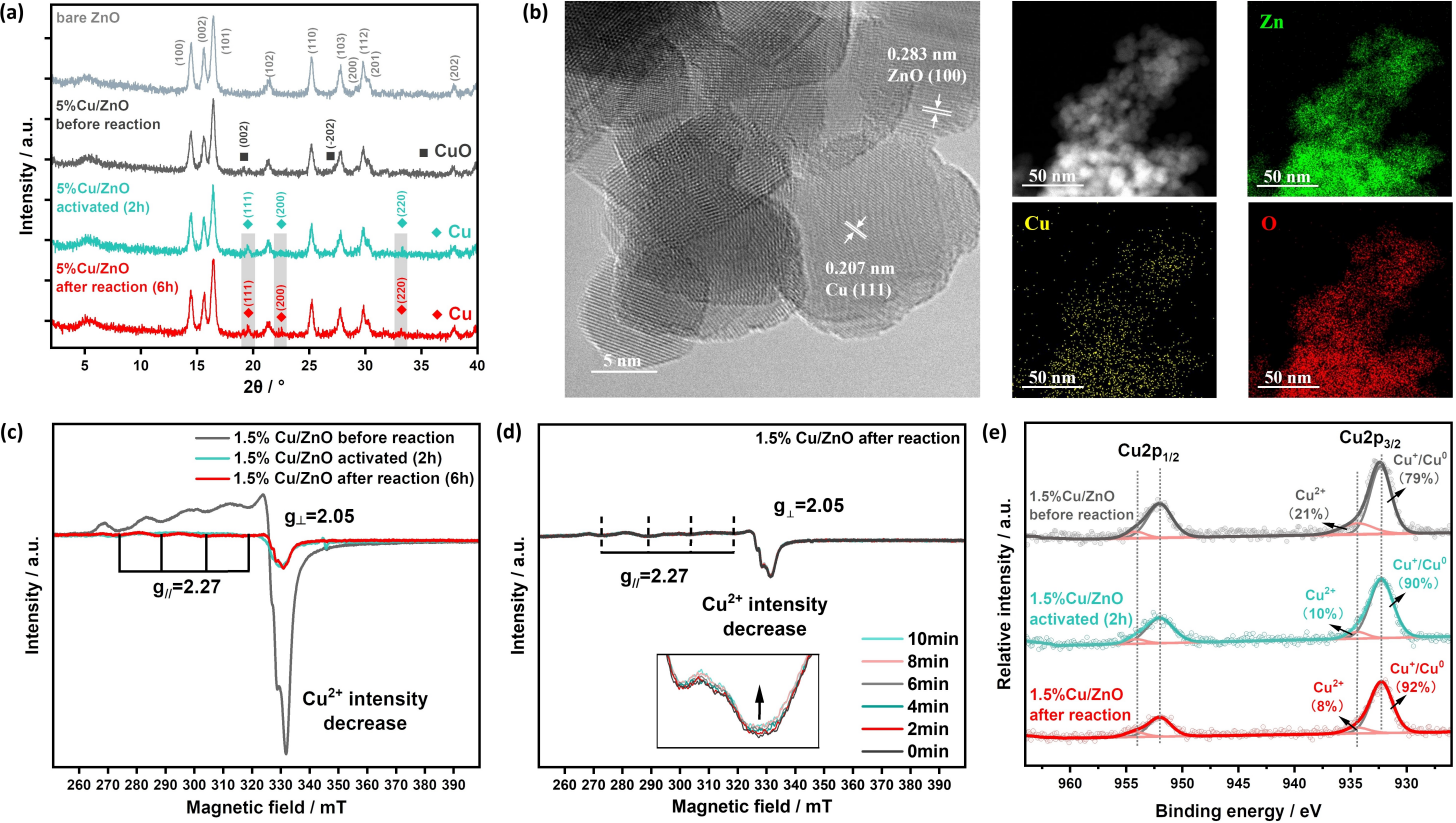
Characterization of photocatalysts. (a) XRD patterns of bare ZnO, 5 %Cu/ZnO samples (before, activated and after reaction). (b) TEM and EDS mapping image of Cu/ZnO after reaction sample. (c) EPR spectra of 1.5 % Cu/ZnO samples (before, activated and after reaction). (d) In situ EPR spectra of 1.5 % Cu/ZnO after reaction sample. (e) Deconvolution of XPS spectra in the Cu 2p_3/2_ region for 1.5 % Cu/ZnO samples (before, activated and after reaction).

A series of characterizations were carried out to obtain structural information on the active Cu/ZnO photocatalyst. As the XRD spectra (Figure S2) displays no extra peaks for the 1.5 % Cu/ZnO sample mentioned above, a higher loading amount (5 wt %) of Cu was used for detailed analysis. As shown in Figure 3a, the peaks assigned to (002) and (−202) lattice planes of crystalline CuO are observed for the sample before a reaction, while only peaks corresponding to (111), (200) and (220) lattice planes of crystalline Cu are observed after 2 h and 6 h irradiation. This result suggests that the Cu species can be reduced in situ by the photoelectrons during the reaction (denoting an activation process) and metallic Cu may be the active catalytic species. Transmission electron microscopy (TEM) and energy dispersive spectroscopy (EDS mapping) were conducted on the post‐reaction Cu/ZnO sample, as shown in Figure 3b. The Cu nanoparticles are homogeneously deposited on the ZnO support. The diameters of ZnO range from 10 to 20 nm, while the diameter of Cu NPs is roughly 10 nm, respectively. These nanoparticles are further identified by the d spacing of fringes, with 0.283 nm for ZnO (100)^[44]^ and 0.207 nm for Cu (111),^[45]^ respectively. The electron paramagnetic resonance (EPR) results (Figure 3c) show a sharp peak for the sample before the reaction, which is owing to I=3/2 of Cu^2+^, so CuO hyperfine structure (g_//_=2.27, A_//_≈90 G) can be observed by EPR spectra, which agrees with the distorted octahedral coordination of Cu^2+^ ions in CuO clusters.[^46^, ^47^, ^48^] The intensity of Cu^2+^ peak decreases after 2 h irradiation and remains similar after 6 h reaction, suggesting that the Cu^2+^ species is likely reduced at the beginning of the photoreaction and the reduced Cu metal is EPR‐silent. Thus, there is an in situ activation process where most of Cu^2+^ is photo‐reduced to copper, which then acts as the reaction sites. A similar trend can also be observed from the EPR results on the 5 % Cu loading samples (Figure S20). Some lattice fringes corresponding to the CuO can also be found with 0.251 nm of (002),^[49]^ as shown in Figure S21. Taking into account the above TEM and EPR analysis, some CuO still remains in the sample after the in situ activation process. In order to further investigate the function of metallic Cu in the system, in situ EPR was conducted on the Cu/ZnO post‐reaction sample as it is similar to the sample after 2 h activation and the results are shown in Figure 3d. A weak Cu^2+^ peak with g_//_=2.27 and A_//_≈90 G can be observed in the EPR spectra, while upon in situ irradiation for 0 min to 10 min, the intensity of this Cu^2+^ peak gradual decreases, suggesting that the metallic Cu may function as electron acceptors during the photocatalytic process. Figure 3e shows the deconvolution of X‐ray photoelectron spectroscopy (XPS) spectra in the Cu 2p_3/2_ region of Cu/ZnO sample before, after 2 h activation and after 6 h reaction. The peak observed at around 933.7 eV can be attributed to CuO, and the other peak that appears at 932.2 eV can be assigned to the mixture of Cu/Cu_2_O.^[50]^ It is clear that the concentration of Cu^2+^ decreases (21 % to 10 %) in the sample after 2 h light irradiation, suggesting the reduction of Cu^2+^ during the activation process, after which the concentration of Cu^2+^ remains around 8 % at the end of reaction, further indicating that the electron acceptors during the photocatalytic process may be metallic Cu. The same sequence is also observed on the 5 % Cu loading sample (32 % to 9 % to 10 %, Figure S22). The above structural characterizations indicate that the active photocatalyst is composed of Cu species and ZnO and the active sites are probably metallic Cu NPs, which act as electron acceptors during the reaction.

**Figure 4:**
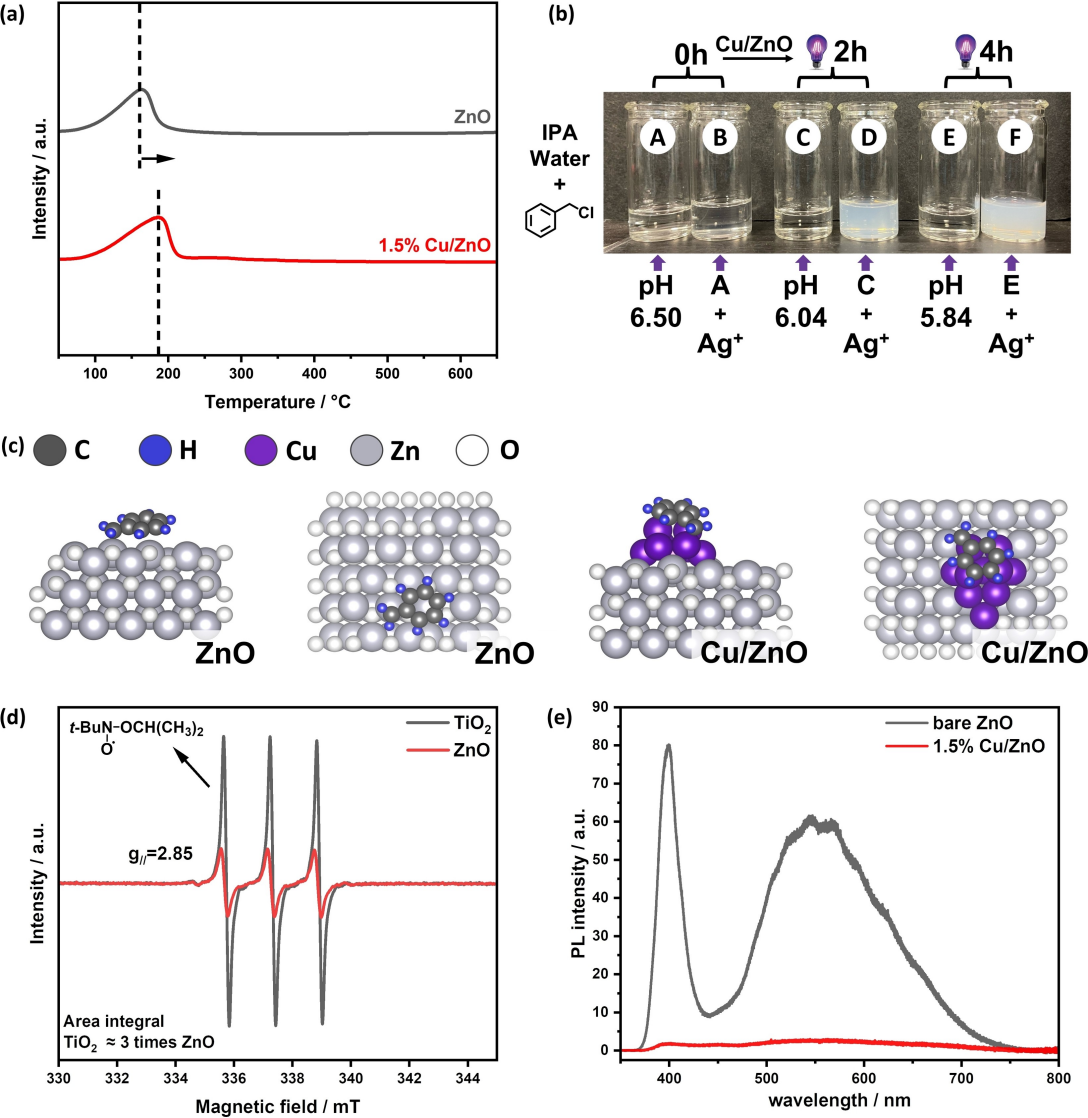
(a) TPD spectra of benzyl chloride on bare ZnO and 1.5 % Cu/ZnO 6 h reaction sample; (b) Images of reaction solutions for identifying reaction intermediates. A: benzyl chloride in isopropanol/water mixture. C and E: photoreduction of benzyl chloride using 1.5 % Cu/ZnO after irradiation by 365 nm LED for 2 h and 4 h, respectively under deaerated conditions. B, D and F: photos after adding the same amount of AgNO_3_ into A, C and E; (c) Optimized adsorption model of benzyl radical on selected surfaces of ZnO and Cu/ZnO for DFT calculation; (d) EPR spectra for capture experiment of alkoxy radicals from 2‐propanol over TiO_2_ and ZnO; (e) PL spectra of bare ZnO and Cu/ZnO after reaction sample.

The first step of the photocatalytic process is the reactant adsorption, so benzyl chloride‐temperature programmed desorption (TPD) was carried out to monitor the reactant desorption on the 6 h reaction sample as it is similar to the 2 h reaction one. From the TPD results (Figure 4a), the desorption peak of benzyl chloride shifts to a higher temperature after the loading of Cu species. This result suggests the existence of a stronger interaction between the catalyst and the reactant, which is induced by the Cu species. The photoabsorption properties of bare ZnO and Cu/ZnO were also investigated by ultraviolet‐visible (UV/Vis) spectroscopy (Figure S23). After introducing copper onto ZnO, the photoabsorption remains similar at the wavelength below 400 nm, indicating the intact band structure of ZnO. In contrast, an enhancement is observed in the range ≥400 nm for the post‐reaction sample. The broadened enhancement throughout the visible range may be attributed to CuO,^[51]^ while the stronger absorption at around 625 nm can be contributed by the localized surface plasmon resonance (LSPR) effect from the Cu NPs.[^52^, ^53^] The band gaps of both bare ZnO and Cu/ZnO have also been calculated to be 3.15 eV from the UV/Vis results (Figure S24).

Control experiments were conducted, and the results are shown in Table S2. In the absence of either light irradiation or Cu/ZnO photocatalyst, no conversion of benzyl chloride and generation of bibenzyl are detected. It has been reported that C−Cl bond activation is an electron‐assisted reduction process.^[54]^ Thus, the electron capture experiment was carried out to examine this reaction pathway of benzyl chloride in our photocatalytic system. After 2 h 365 nm LED irradiation, there is no conversion of benzyl chloride observed with the addition of electron scavenger NaIO_3_ (Figure S25). These results suggest the homo‐coupling of benzyl chloride is indeed a photocatalytic process requiring the presence of photoelectrons generated by light irradiation on a photocatalyst. The pathway of the C−Cl decomposition was further investigated by chemical precipitation, as shown in Figure 4b. Initially, the transparent reaction solution of benzyl chloride in the mixture of 2‐propanol and water solution remains colourless in the absence of Cu/ZnO catalyst (bottle A) and in the presence of Cu/ZnO catalyst under 365 nm LED irradiation for 2 h (bottle C), and 4 h (bottle E), respectively. Upon adding AgNO_3_ dropwise into bottle C and bottle E, white precipitates appear in both bottles (bottle D and bottle F), which suggests the existence of Cl anions (Cl^−^) in the solution. Moreover, it is reasonable to observe more white precipitates in bottle F (4 hours) than in bottle D (2 hours), as a longer reaction time results in higher Cl^−^ concentration. In contrast, bottle B remains transparent after adding AgNO_3_, excluding the existence of Cl^−^ without the photoreaction. The production of Cl anions often leads to the formation of HCl in the solution, thus the pH was also monitored. Expectedly, a decreasing trend in pH is obtained with increasing reaction time from 0 h to 4 h (pH: 6.5>6.04>5.84). This result indicates that the C−Cl group cleavage is the important step.

Both the precursor PhCH_2_Cl and the key intermediate PhCH_2_⋅ on both bare ZnO(100) and Cu_10_/ZnO(100) surfaces (Figure S26 and 4c, respectively) were then studied by density functional theoretical (DFT) calculations (the calculation details listed in the Supporting Information). Both bare ZnO(100) and Cu_10_/ZnO(100) surfaces attracts the precursor PhCH_2_Cl (Table S3). In particular, the introduction of copper loading can significantly enhance the adsorption of benzyl radical PhCH_2_⋅ on Cu decorated ZnO(100) (−2.39 eV on Cu_10_/ZnO(100) and −1.19 eV on ZnO(100), suggesting an enhanced stabilization of the intermediate on the surface of the catalyst, which is crucial for the further coupling reaction to produce bibenzyl with high selectivity. TiO_2_, as a most widely‐used semiconductor with a similar band structure,^[55]^ was used to compare with ZnO. It should be noted that Cu/TiO_2_ has been reported to be an efficient photocatalyst in driving homo‐coupling of a relatively easy process of benzyl bromide with a good yield.^[33]^ However, it is reported that Cu/TiO_2_ exhibits a very moderate yield (53 %) and selectivity (57 %) towards the coupling product with the corresponding chloride substrate. As a reference, the adsorption energy of PhCH_2_⋅ on Cu_10_/TiO_2_(101) was also calculated by DFT (Figure S26). Compared to Cu_10_/ZnO(100) (−2.39 eV), the adsorption energy of benzyl radicals on Cu_10_/TiO_2_(101) is comparable (−2.37 eV, Table S3), suggesting that apart from the adsorption/desorption of benzyl radicals, there is another factor to dominate the bibenzyl selectivity. According to kinetic studies of 2‐propanol adsorption on ZnO and TiO_2_, the adsorption capacity of 2‐propanol in dark on TiO_2_ is 6.3 times higher than on ZnO.^[56]^ Meanwhile, the photo‐oxidation rate of 2‐propanol under UV irradiation on TiO_2_ is 2.6 times higher than on ZnO.^[57]^ Furthermore, we studied the photo‐oxidation rate of 2‐propanol on both TiO_2_ and ZnO, and the capture experiment of alkoxy radicals produced from 2‐propanol was then conducted, and the results are shown in Figure 4d. 2‐nitroso‐2‐niethylpropane (*t*‐BuNO) was used as the trapping agent following the reaction equation S3[^58^, ^59^] The trapped nitroxides show strong signals (g_//_=2.85, A≈60 G) on the EPR spectrum after 30 s irradiation, and the integral of nitroxides generated on TiO_2_ is roughly 3 times higher than that on ZnO, which is similar to the reference result (2.6 times), indicating a faster photo‐oxidation of 2‐propanol on TiO_2_ surface. Therefore, after the formation of alkoxy radicals due to the reaction between photoholes and 2‐propanol, the amount of the surface adsorbed H on TiO_2_ would be much higher than that on ZnO, resulting in the formation of dehalogenation byproduct (toluene). The temporal study on Cu/TiO_2_ (Figure S27) indeed shows that after 1 h irradiation, Cu/TiO_2_ exhibits nearly 80 % selectivity towards bibenzyl. However, this selectivity continuously decreases in the following 2 h irradiation and maintains around 57 % afterwards. The gradually increasing amount of surface adsorbed H on Cu/TiO_2_ could be responsible for this decrease. By the end of experiment, almost 40 % of benzyl radicals react with the surface adsorbed H to generate toluene. In contrast, the selectivity to bibenzyl remains as high as 90 % with negligible formation of toluene during the term of 6 h reaction when Cu/ZnO is used as the photocatalyst. These results indicate that the moderate photo‐oxidation half reaction is also critical in achieving high selectivity of bibenzyl from benzyl chloride.

Finally, besides the enhanced adsorption of the reactant by Cu and moderate oxidation ability of ZnO, the charge transfer on ZnO and Cu/ZnO was also investigated by photoluminescence (PL) spectroscopy (Figure 4e). There are two peaks observed in the PL spectra of bare ZnO. The strong peak at around 400 nm in the ultraviolet (UV) region is attributed to the near‐band‐edge emission of ZnO, and the broad peak at around 550 nm can be assigned to the oxygen vacancies‐related emission.^[60]^ A remarkable decrease of both emissions in PL intensity is observed on Cu/ZnO compared to bare ZnO, indicating the effective inhibition of recombination of charge carriers by the copper loading. Mulliken population analysis was also performed to study the charge transfer direction (Table S4, detailed calculation can be found in the Supporting Information). Benzyl chloride on electrically neutral Cu_10_ cluster or on Cu_10_ cluster with one extra electron, Cu_10_^−^, were optimized. When benzyl chloride adsorbs on the Cu_10_^−^ cluster, −0.111 e of the Mulliken charge of benzyl chloride indicates the electron transfer from Cu_10_^−^ cluster to benzyl chloride. Also, compared to the Cu_10_ cluster, the Mulliken charge on the Cu_10_^−^ cluster is more negative (−0.111 e<−0.086 e), suggesting that the negatively charged Cu cluster can benefit the activation of benzyl chloride.^[61]^

### Reaction Mechanism

**Scheme 1:**
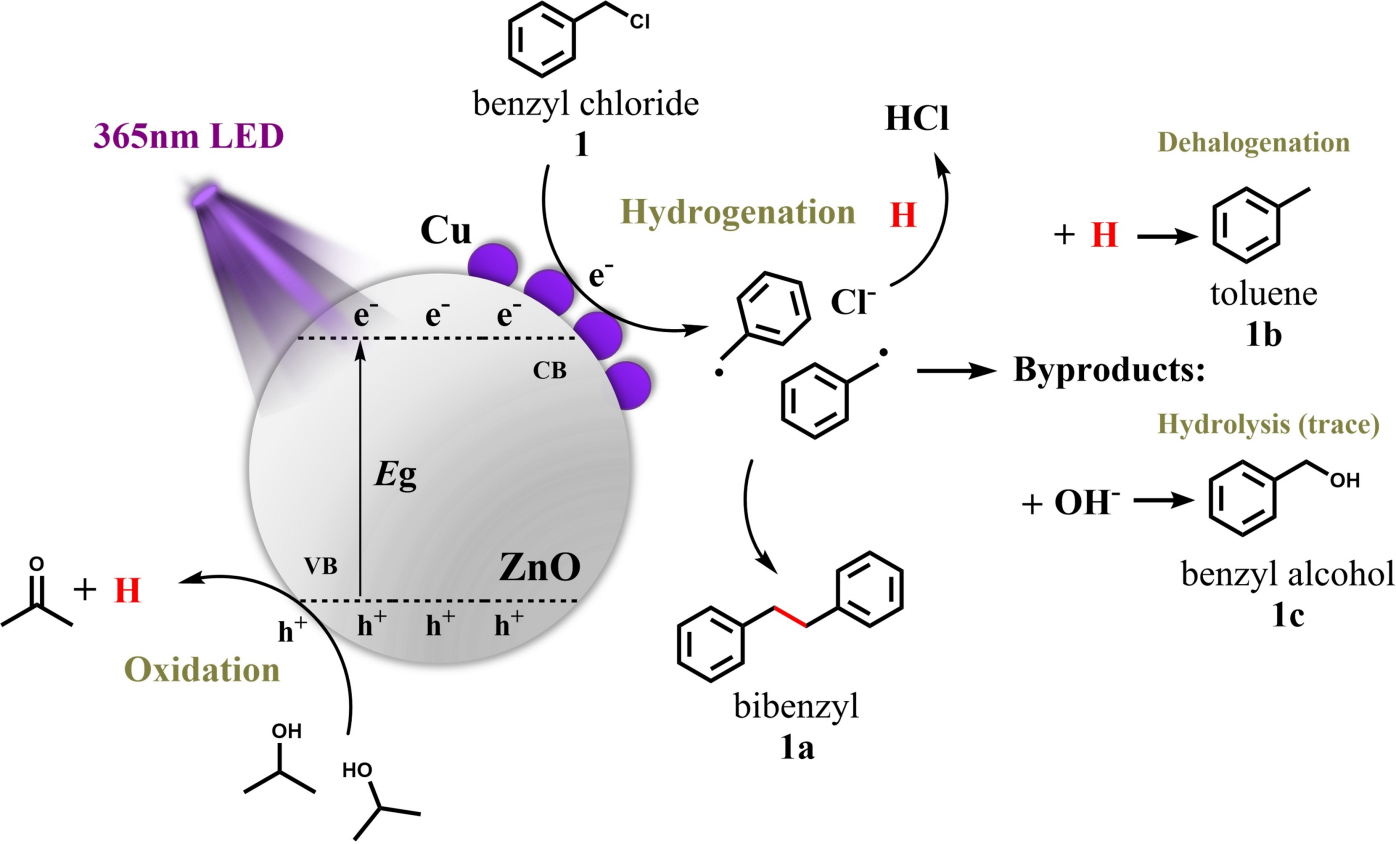
Proposed mechanism for photocatalytic homo‐coupling of benzyl chloride over the Cu/ZnO catalyst.

Based on the results mentioned above, the possible reaction mechanism is proposed and illustrated in Scheme 1. The ZnO semiconductor is firstly excited by the irradiation of 365 nm LED, and the photo‐generated electrons would then migrate to the surface of ZnO. The CuO NPs decorated on the surface are in situ reduced to Cu^+^/Cu by the electrons, which is the activation process. Then this metallic copper loading can not only significantly alleviate the recombination between photo‐generated electrons and holes by working as an electron sink but also enhance the adsorption of benzyl chloride onto the catalyst surface. The adsorbed benzyl chloride accepts electrons from Cu and breaks its C−Cl bond, and 2‐propanol acts as an electron donor, which is moderately oxidized by the photo‐generated holes on the valence band of ZnO and provides limited amount of protons. Those benzyl radicals can be stabilized on the surface of Cu, thus coupling with each other to generate bibenzyl (**1** **a**), while the released Cl atoms in water would form HCl, resulting in a decreased pH. Meanwhile, benzyl radical can either react with protons to generate toluene (**1** **b**) or hydroxy radical to generate trace amounts of benzyl alcohol (**1** **c**). The high selectivity to 1a is due to the abundant formation of benzyl radicals without excessive presence of protons.

## Conclusion

In summary, thanks to bibenzyl as the major raw material for pharmaceutical synthesis and the higher natural abundance of organic chlorides over others, e.g., bromides, the coupling of benzyl chlorides is highly sought‐after. However, high selectivity and high yield towards bibenzyl using chlorides have not been achieved by heterogeneous photocatalysis because of the strong C−Cl bonds. This work reports a strategy for heterogeneous photocatalytic homo‐coupling of benzyl chlorides, leading to the first successful case with a 92 % yield. A series of transition metal co‐catalysts were initially screened and the reaction conditions (including solvents, Cu loading and water content) were then optimized. The unprecedented high yield towards bibenzyl can be achieved over the Cu/ZnO photocatalyst with 1.5 wt % copper loading using 2‐propanol and water mixture as the solvent. Furthermore, the photocatalytic activity is very stable during the term of 8 cycles without noticeable decay in the yield of bibenzyl. The Cu/ZnO photocatalyst can also be applied to 11 benzyl chloride and derivatives to achieve their homo‐couplings, of which 10 derivatives have gained excellent yields (72–96 %). This shows that electron‐donating groups favor this synthetic process and our catalyst represents a strong potential for a wide application. Fundamentally, the TPD result indicates Cu can improve the interaction between benzyl chloride and the catalyst. By comparing Cu/ZnO with Cu/TiO_2_ using EPR measurement, we found that photo‐oxidation rate of 2‐propanol on TiO_2_ is 3 times higher than that on ZnO, indicating the moderate oxidation ability of ZnO is another factor in achieving the high bibenzyl yield from chloride substrates.

## Conflict of interest

The authors declare no conflict of interest.

1

## Supporting information

As a service to our authors and readers, this journal provides supporting information supplied by the authors. Such materials are peer reviewed and may be re‐organized for online delivery, but are not copy‐edited or typeset. Technical support issues arising from supporting information (other than missing files) should be addressed to the authors.

Supporting Information

## Data Availability

The data that support the findings of this study are available from the corresponding author upon reasonable request.
