# Supplementary material for: Effective Activation of Strong C−Cl Bonds for Highly Selective Photosynthesis of Bibenzyl via Homo‐Coupling
Source: Angew Chem Int Ed Engl. 2023 Sep 18;62(43):e202307907. doi: 10.1002/anie.202307907 (PMC10952150; doi:10.1002/anie.202307907)
Supplement: Supplementary file 1 — Supporting Information [file ANIE-62-0-s001.pdf]

Angewandte Chemie
Eine Zeitschrift der Gesellschaft Deutscher Chemiker

Supporting Information

Effective Activation of Strong C–Cl Bonds for Highly Selective Photosynthesis of Bibenzyl via Homo-Coupling

*Q. Yang, X. Li, L. Chen, X. Han, F. R. Wang, J. Tang**

Table of Contents

Table of Contents 2
Experimental Procedures.. 3
Additional Characterizations.. 7
References 27

Experimental Procedures

Synthesis of ZnO: The ZnO NPs were prepared by a precipitation method[1], where 0.01 mol $Zn(NO_3)_2$ and 0.01 mol oxalic acid were separately dissolved in 100 ml deionised water in two beakers at room temperature. The oxalic acid solution was then dropwise added into $Zn(NO_3)_2$ solution to obtain zinc oxalate precipitates. After the precipitates were washed and filtered, the dried powders were calcined at 350 °C in air for 6 h.

Synthesis of metal/ZnO photocatalyst:

I: Cu, Pd, Ag, In, Rh, Au, Pt, Ru, Ir NPs were preparead on ZnO via a photo-deposition method. In particular, the deposition of Cu on ZnO is described as the following:

200 mg of ZnO powders were dispersed into a methanol water mixture (10 ml methanol and 30 ml water), certain amount of $Cu(NO_3)_2 \cdot 2.5H_2O$ (0.1, 0.5, 1, 1.5, 2 wt% of Cu) was added into the reactor under continuous stirring. The reactor was sonicated for 10min to form a homogeneous suspension and then degassed by argon for 15min to remove dissolved O_2. The purged reactor was later irradiated under 365 nm LED for 2 h in a multi-channel reactor. After irradiation, the reacted suspension (Cu/ZnO) was filtered and washed by deionised water for 3 times, and eventually dried at 60 °C in a vacuum oven.

For the deposition of 1.5 wt% Pd, Ag, In, Rh, Au, Pt, Ru and Ir, $PdCl_2$ anhydrous, $AgNO_3$, $InCl_3$, $RhCl_3 \cdot 3H_2O$, $HAuCl_4 \cdot 4H_2O$, H_2PtCl_6, $RuCl_3 \cdot xH_2O$ and $IrCl_3 \cdot xH_2O$ were used as the metal source, respectively and other procedure is identical to the Cu loading.

II: Ni, Fe, Co were prepared on ZnO via an impregnation method: certain amount of $Ni(NO_3)_2 \cdot 6H_2O$ ($Co(NO_3)_2 \cdot 6H_2O$ and $Fe(NO_3)_3 \cdot 9H_2O$ for Co and Fe) was firstly dissolved in 500 µl deionised water. 200 mg ZnO was then added to the above solution under vigorous stirring in an alumina crucible. After evaporating water, the crucible was transferred to a muffle furnace and calcined at 400 °C for 2 h with a ramping rate of 5 °C/min.

Photocatalytic activity tests:

Firstly, 10 mg Cu/ZnO photocatalyst was dispersed into 5 ml solution (2-propanol: water = 1:1) containing 10 mM benzyl chloride, and the solution was then sonicated for 5min to form a homogeneous suspension. The suspension was purged by Argon for 10 min to remove dissolved O_2. The reactor containing the suspension was eventually placed into a PCX-50C multi-channel photochemical reaction system (Beijing Perfectlight, Figure S1) equipped with 365 nm LEDs and irradiated for 6 h under continuously stirring condition at room temperature (temperature was controlled by a cooling bath thermostat). The solid photocatalyst was filtered and then the liquid mixture was analysed by Shimadzu GC-MS-QP2010SE using Rxi-5SilMS Column.

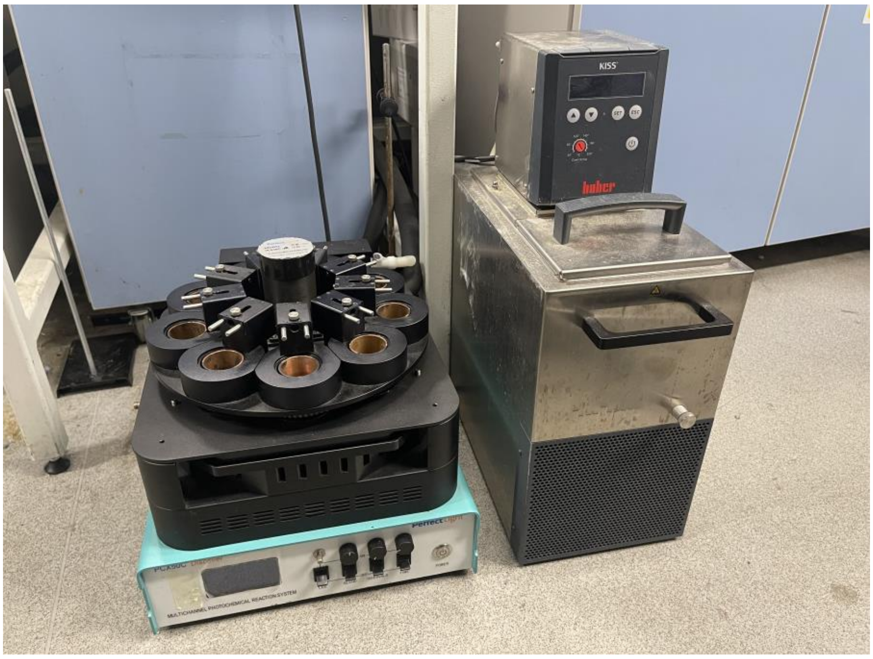

Figure S1 PCX-50C multi-channel photochemical reaction system (Beijing Perfectlight) and cooling bath thermostat.

Table S1. Performance of bibenzyl synthesis over different photocatalysts.

Entry	Catalyst	Metal concentration	Con. (%)	Sel.1a (%)	Sel.1b (%)	Sel.1c (%)
1	ZnO	-	-	-	-	-
2	Co/ZnO	1.5%	-	-	-	-
3	Fe/ZnO	1.5%	-	-	-	-
4	Ir/ZnO	1.5%	3%	26%	40%	34%
5	Ru/ZnO	1.5%	6%	37%	34%	29%
6	Ni/ZnO	1.5%	7%	53%	12%	35%
7	Au/ZnO	1.5%	9%	44%	31%	25%
8	Pt/ZnO	1.5%	12%	18%	65%	17%
9	Rh/ZnO	1.5%	22%	31%	62%	7%
10	In/ZnO	1.5%	38%	75%	13%	12%
11	Ag/ZnO	1.5%	94%	31%	68%	1%
12	Pd/ZnO	1.5%	96%	49%	44%	4%
13	Cu/ZnO	1.5%	97%	93%	3%	2%
14	Cu/ZnO	0.1%	86%	78%	7%	8%
15	Cu/ZnO	0.5%	88%	86%	3%	2%
16	Cu/ZnO	1%	91%	87%	4%	2%
17	Cu/ZnO	2%	98%	86%	3%	2%
18	Cu/ZnO	2.5%	99%	84%	3%	3%

Reaction conditions: 10mM reactant, 10 mg photocatalyst in 5 ml solvent (2-propanol: water = 1:1) under 365 nm LED irradiation for 6 h in Ar at room temperature.

Reaction equations:

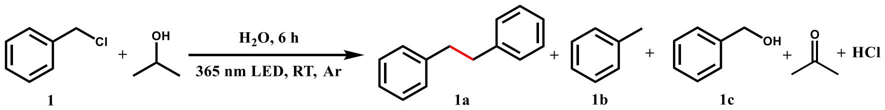

Equation S1 overall equation for photocatalytic homo-coupling of benzyl chloride

$$conversion = \frac{mole\ of\ benzyl\ chloride\ reacted}{moles\ of\ benzyl\ chloride\ fed\ into\ the\ system} \times 100\%$$

$$selectivity = \frac{mole\ of\ the\ target\ product\ generated}{mole\ of\ benzyl\ chloride\ reacted} \times 100\%\ (times\ 2\ for\ bibenzyl)$$

$$yield = conversion \times\ selectivity$$

Equation S2 equations for calculating conversion, selectivity and yield

$$(CH_3)_2CHO^{\bullet} \quad + \quad t\text{-BuNO} \longrightarrow t\text{-BuN}(\dot{O})\text{–}OCH(CH_3)_2$$

Equation S3 representation of capturing alkoxy radicals from 2-propanol using t-BuNO

AQE calculation:

The AQE (φ) has been calculated following the formula reported previously[2]. For all measurements, the irradiation window diameter was 4.6 cm and the incident light intensity was measured to be 27.55 $mW \cdot cm^{-2}$. The bibenzyl production amount was 8.76 μmol after 10 min irradiation (600 s). The detailed calculation is shown below:

$$I = \frac{E\lambda}{hc} \times S = \frac{27.55 \times 10^{-3}\ \mathrm{W \cdot cm^{-2}} \times 600\ \mathrm{s} \times 365 \times 10^{-9}\ \mathrm{m}}{6.626 \times 10^{-34}\ \mathrm{J \cdot s} \times 3 \times 10^{8}\ \mathrm{m \cdot s^{-1}}} \times 3.14 \times 2.3\ \mathrm{cm} \times 2.3\ \mathrm{cm} = 5.04 \times 10^{20}$$

Where E is the energy of photons, λ is the wavelength, h is Plank's constant, c is the speed of light, and S is the irradiated area.

$$\varphi = \frac{nR}{I} = \frac{2 \times 8.76 \times 10^{-6} \times 6.02 \times 10^{23}}{5.04 \times 10^{20}} \times 100 = 2.01\%$$

The radical propagation process may lead to an AQE value higher than 100% since some processes can happen without the assistance of photons. In contrast, the AQE is far away from 100% in our case, which suggests that there is not convincing evidence for the radical propagation process. To clarify a radical propagation process in this reaction, futher studies (eg. isotopic experiment and radical capture experiment etc) will be carried out in future. The low AQE value also indicates the challenge for C-Cl bond activation.

Control experiments:

Control experiments were conducted, and the results are shown below in Table S1

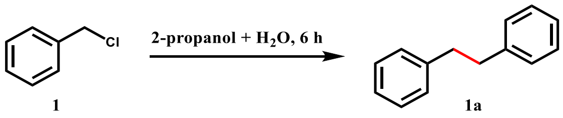

Table S2. Control experiments for photocatalytic Ullmann homo-coupling of benzyl chloride.

Entry	Conditions	Yield of **1a** / %
1	No light	-(N.D.)
2	No Cu/ZnO	-(N.D.)

Reaction conditions: 10mM reactant, 10 mg photocatalyst in 5 ml solvent (2-propanol: water = 1:1) under 365 nm LED irradiation for 6 h, Ar, room temperature.

DFT calculation:

The Vienna Ab Initio Package (VASP)[3],[4] was used to perform all the density functional theory (DFT) calculations within the generalised gradient approximation (GGA) using the PBE[5] formulation. The projected augmented wave (PAW) potentials were used [6],[7] to describe the ionic cores and take valence electrons into account using a plane-wave basis set with a kinetic energy cut-off of 450 eV. Partial occupancies of the Kohn−Sham orbitals were allowed using the Gaussian smearing method and a width of 0.05 eV. The on-site corrections (DFT+U) have been applied to the 3d electron of Ti atoms (U_{eff}=4.5 eV) by the approach from Dudarrev et al.[8] The electronic energy was considered self-consistent when the energy change was smaller than 10^{-5} eV. Geometry optimisation was considered convergent when the force change was smaller than 0.02 eV/Å. Grimme's DFT-D3 methodology[9] was used to describe the dispersion interactions.

The equilibrium lattice constants of wurtzite ZnO unit cell were optimised to be a=b=3.272 Å and c=5.291 Å when using a 15×15×9 Monkhorst-Pack k-point grid for Brillouin zone sampling. It was then used to construct a ZnO(100) surface model with $p(4\times2)$ periodicity in the X and Y directions and three stoichiometric layers in the Z direction by vacuum depth of 15 Å in order to separate the surface

slab from its periodic duplicates. In another model, a Cu_{10} cluster resides onto ZnO(100). During structural optimisations, a 2×2×1 in the Brillouin zone was used for k-point sampling, and the bottom stoichiometric layer was fixed while the rest were allowed to relax fully.

The equilibrium lattice constants of anatase TiO_2 unit cell were optimised to be a=b=3.858 Å and c=9.652 Å when using a 10×10×4 Monkhorst-Pack k-point grid for Brillouin zone sampling. It was then used to construct a TiO_2(101) surface model with *p*(1×3) periodicity in the X and Y directions and two stoichiometric layers in the Z direction by vacuum depth of 15 Å in order to separate the surface slab from its periodic duplicates. A Cu_{10} cluster resides onto TiO_2(101). During structural optimisations, a 2×1×1 in the Brillouin zone was used for k-point sampling, and the bottom stoichiometric layer was fixed while the rest were allowed to relax fully.

The adsorption energy (E_{ad}) was defined as

$$E_{ad} = E_{total} - E_{sub} - E_{adsorbent}$$

where E_{total}, E_{sub} and $E_{adsorbent}$ is the energy of the adsorbate adsorbed on the polyimide, the energy of clean polyimide, and the energy of adsorbent molecule in a cubic periodic box with a side length of 20 Å and a 1×1×1 Monkhorst-Pack k-point grid for Brillouin zone sampling, respectively.

DFT for Mulliken population analysis

The DFT calculation was performed with the Gaussian 16 package using the hybrid B3LYP functionals. The 6-311++G** basis set was used for C, H and Cl elements, and SDD pseudo potential basis set for Cu element[10,11].

Additional Characterizations

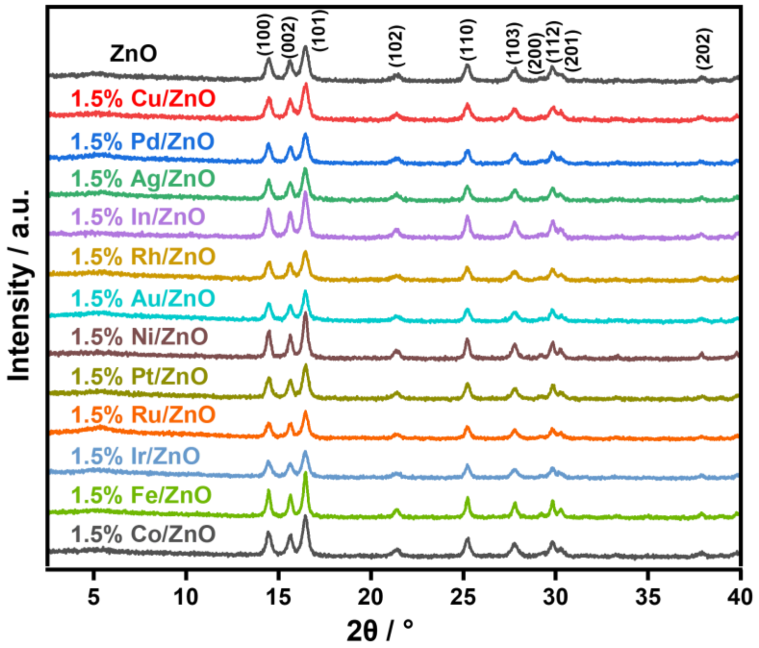

Figure S2 XRD patterns of bare ZnO, 1.5% M/ZnO (M= Cu, Pd, Ag, In, Rh, Au, Ni, Pt, Ru, Ir, Fe, Co) samples

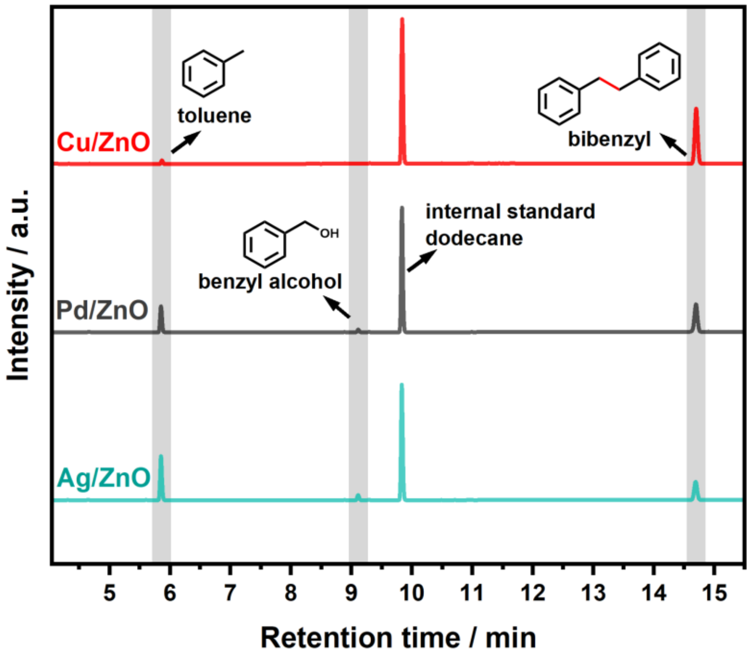

Figure S3 The product GC spectra for 1.5% M/ZnO (M= Cu, Pd, Ag)

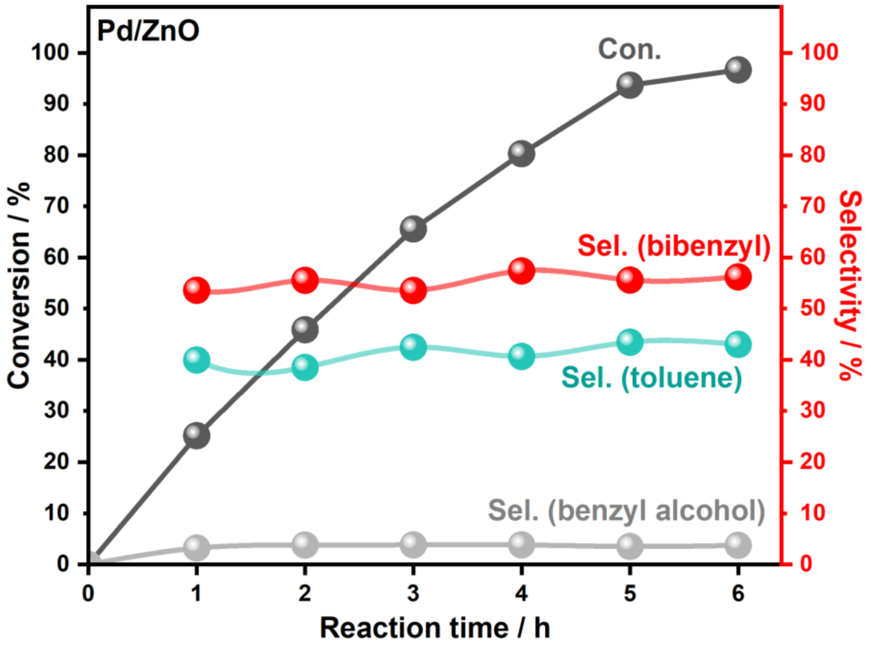

Figure S4 Temporal study of photocatalytic benzyl chloride conversion using Pd/ ZnO. Reaction conditions: 10mM reactant, 10 mg photocatalyst in 5 ml solvent (2-propanol: water = 1:1) under 365 nm LED irradiation for 6h, Ar, room temperature

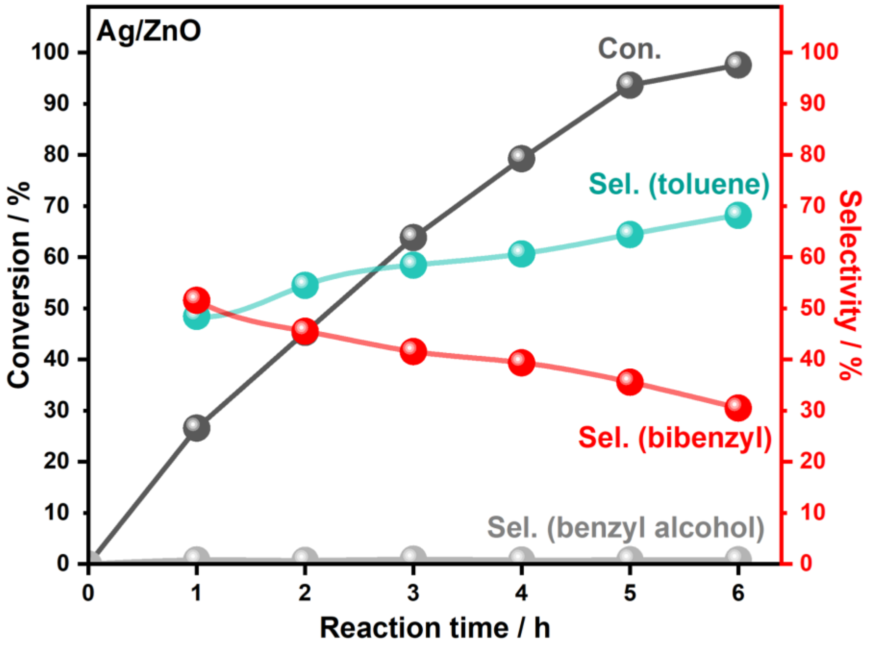

Figure S5 Temporal study of photocatalytic benzyl chloride conversion using Ag/ ZnO. Reaction conditions: 10mM reactant, 10 mg photocatalyst in 5 ml solvent (2-propanol: water = 1:1) under 365 nm LED irradiation for 6h, Ar, room temperature

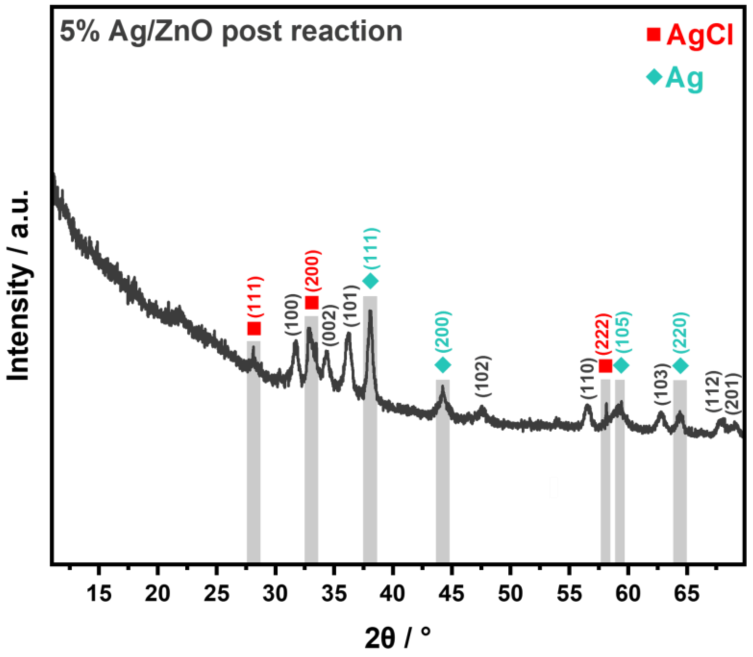

Figure S6 XRD patterns of 5% Ag/ZnO after reaction sample

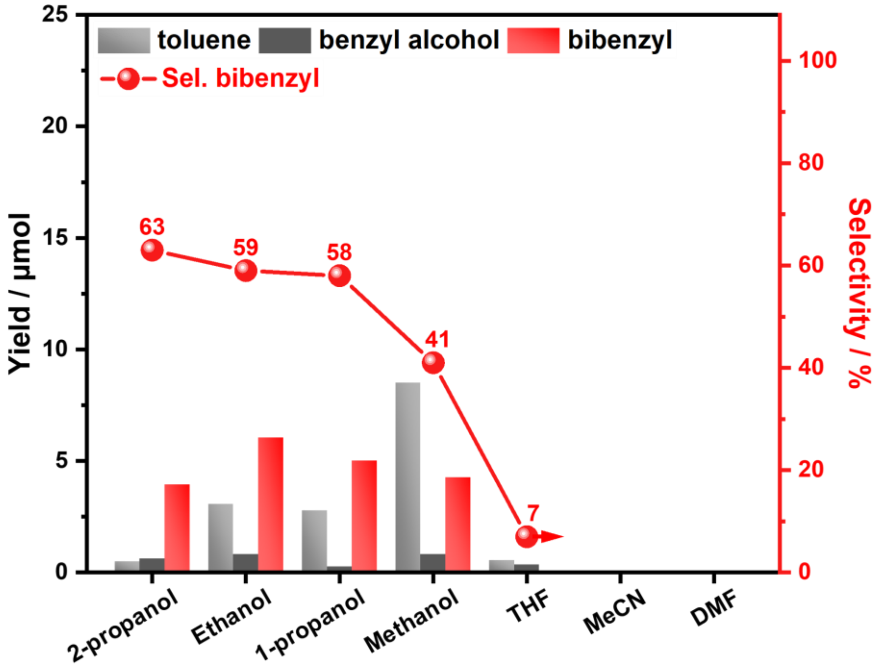

Figure S7 Solvent effect on the photocatalytic coupling of benzyl chloride. Reaction conditions: 10mM reactant, 10 mg photocatalyst in 5 ml pure solvent under 365 nm LED irradiation for 2h in Ar at room temperature

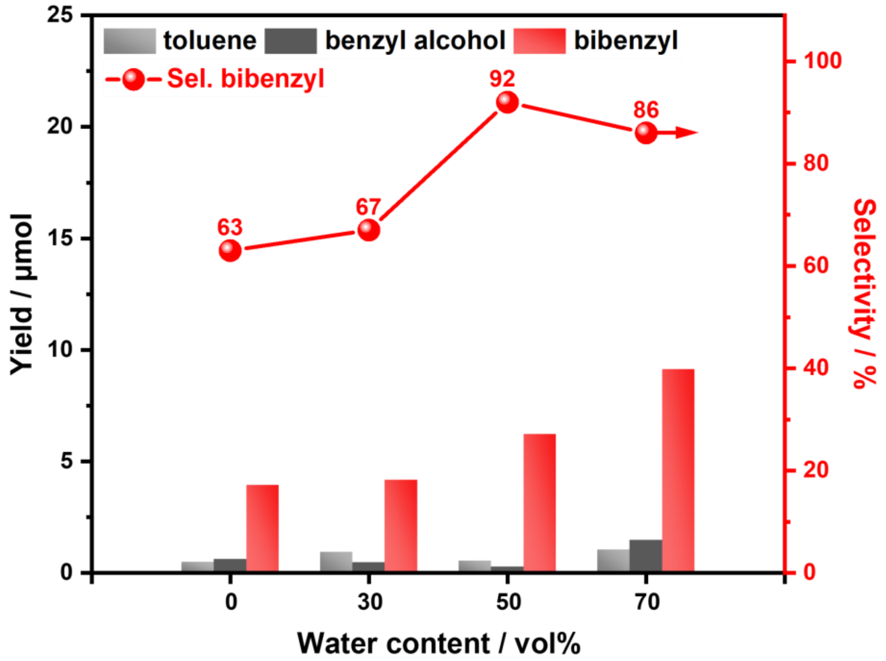

Figure S8 Water contents on the photocatalytic coupling of benzyl chloride. Reaction conditions: 10mM reactant, 10 mg photocatalyst in 5 ml solvent (2-propanol with different water contents) under 365 nm LED irradiation for 2h in Ar at room temperature

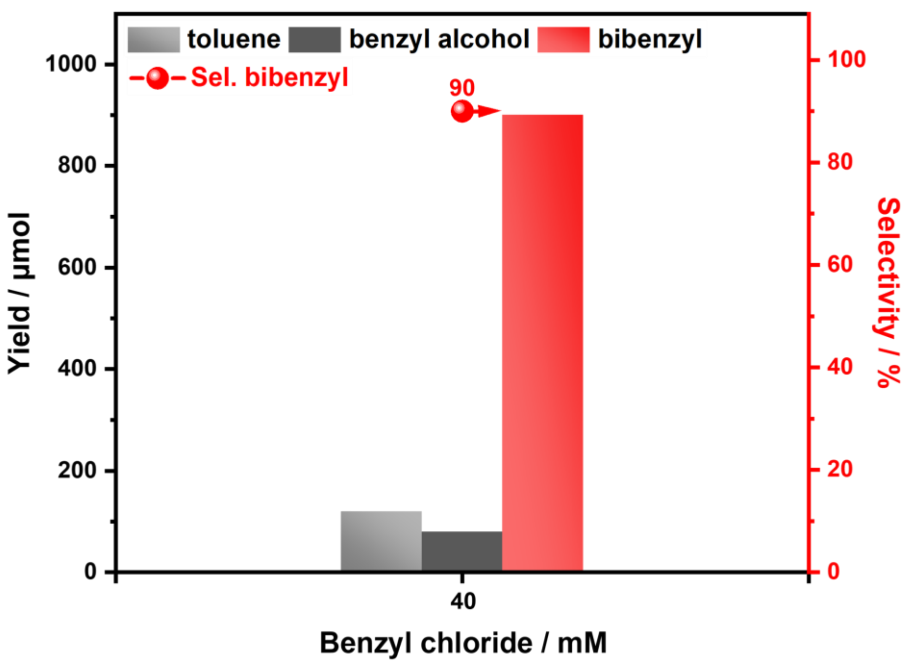

Figure S9 Scaling-up experiment with higher benzyl chloride concentration. Reaction conditions: 40mM reactant, 50 mg photocatalyst in 50 ml solvent (2-propanol: water = 1:1) under 365 nm LED irradiation for 40 h, Ar, room temperature.

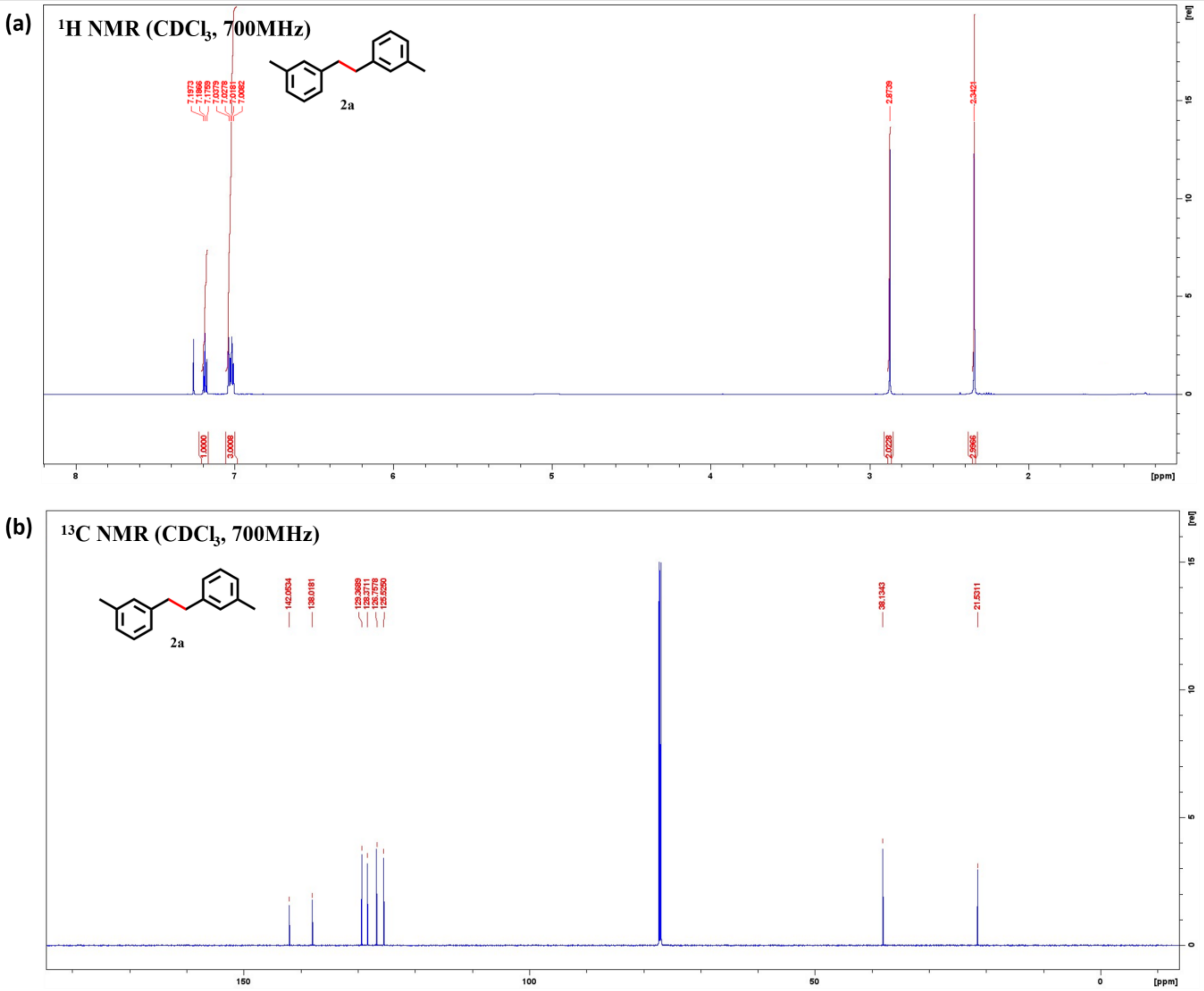

Figure S10 (a) ^{1}H NMR of 2a, and (b) ^{13}C NMR of 2a

^{1}H NMR (700 MHz, $CDCl_3$): δ = 2.34 (s, 6H), 2.88 (s, 4H), 7.02 (m, 6H), 7.19 (m, 2H)
^{13}C NMR (700 MHz, $CDCl_3$): δ 21.5, 38.1, 125.5, 126.7, 128.3, 129.3, 138.0, 142.0

SUPPORTING INFORMATION

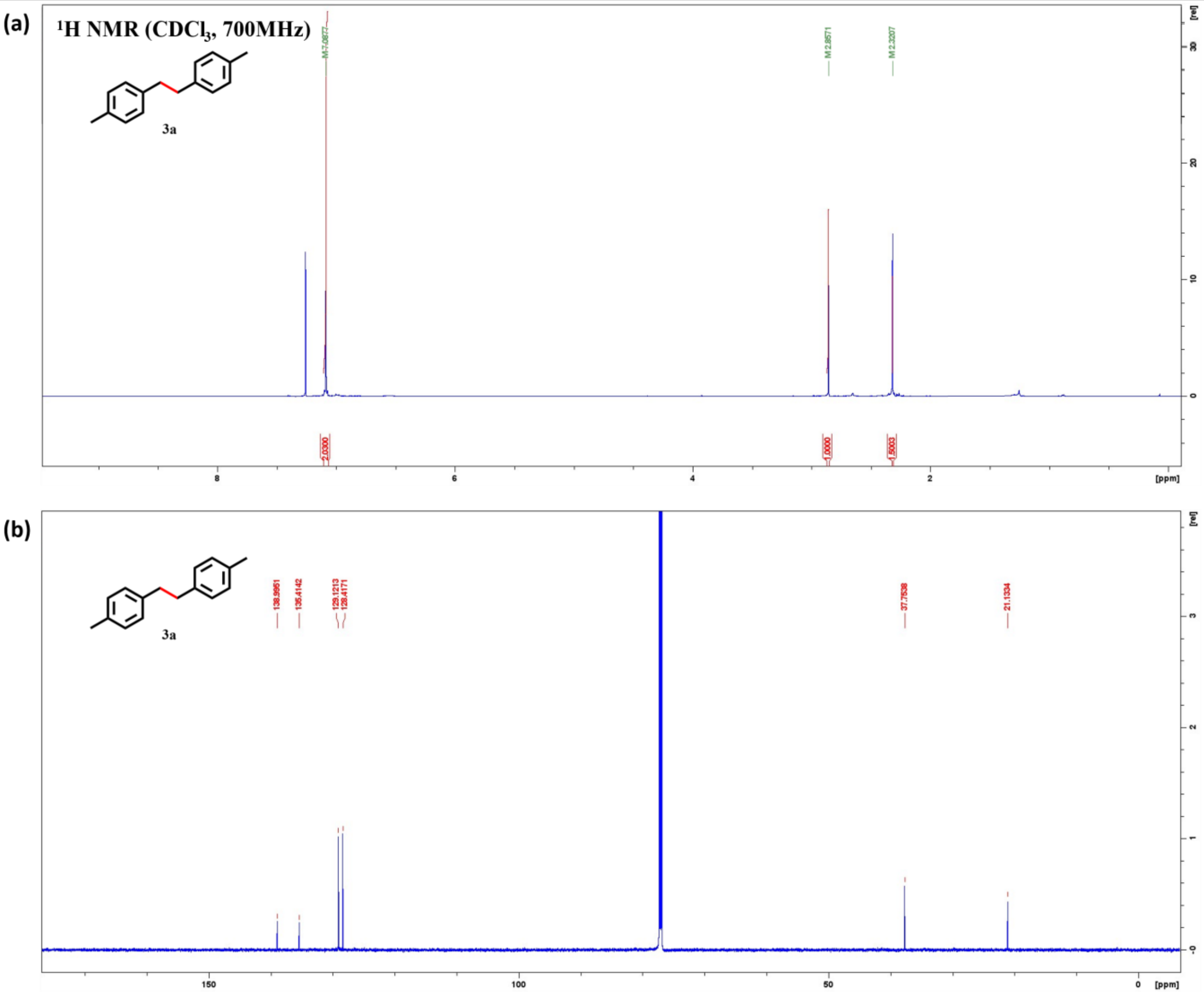

Figure S11 (a) ^{1}H NMR of 3a, and (b) ^{13}C NMR of 3a

^{1}H NMR (700 MHz, $CDCl_3$): δ = 2.32 (s, 6H), 2.86 (s, 4H), 7.09 (s, 8H)
^{13}C NMR (700 MHz, $CDCl_3$): δ 21.1, 37.3, 128.4, 129.1, 135.4, 138.9

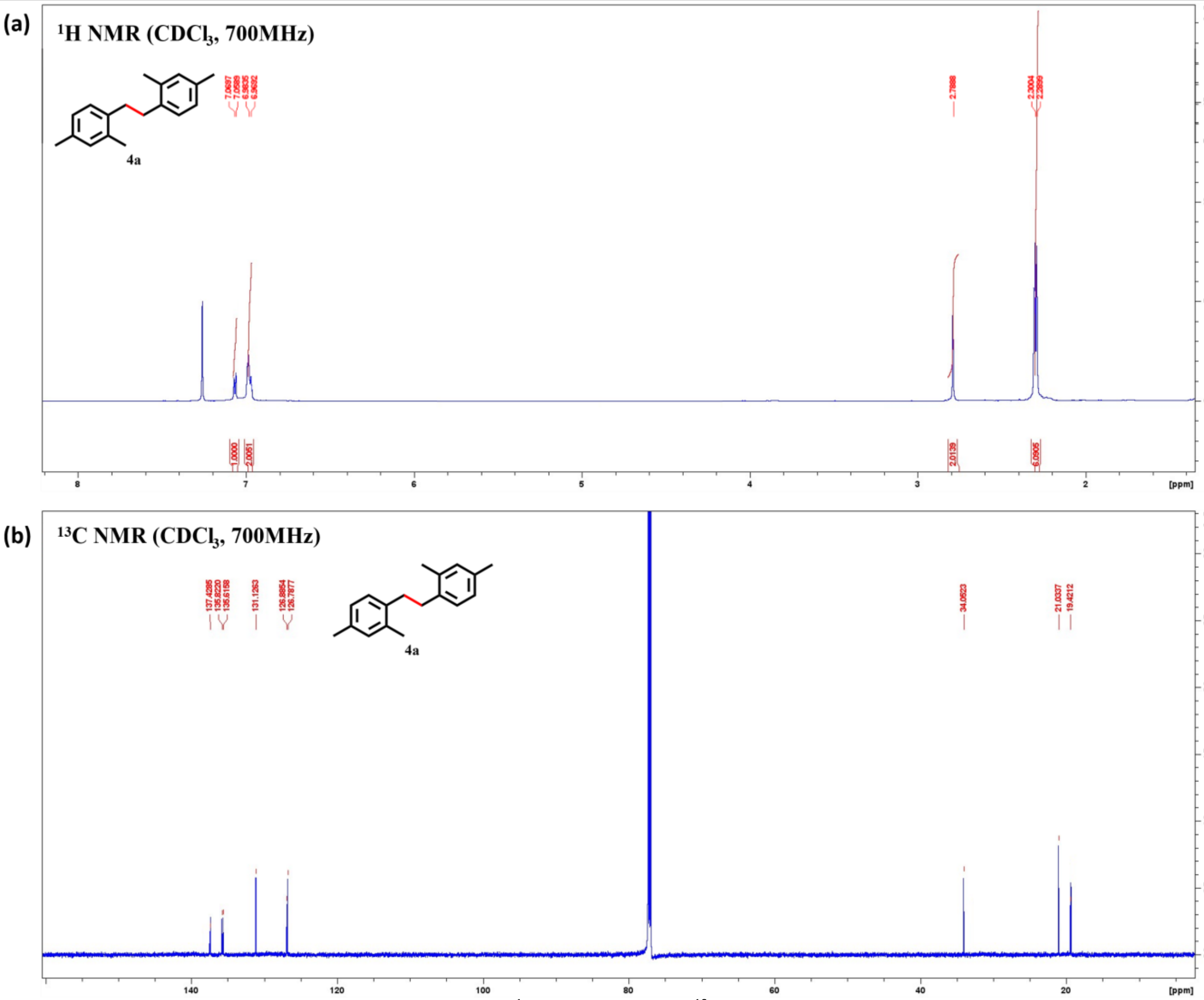

Figure S12 (a) ^{1}H NMR of 4a, and (b) ^{13}C NMR of 4a

^{1}H NMR (700 MHz, $CDCl_3$): δ = 2.35 (s, 12H), 2.83 (s, 4H), 7.01 (s, 6H)
^{13}C NMR (700 MHz, $CDCl_3$): δ 19.4, 21.0, 34.1, 128.8, 128.9, 131.1, 135.6, 135.8, 137.4

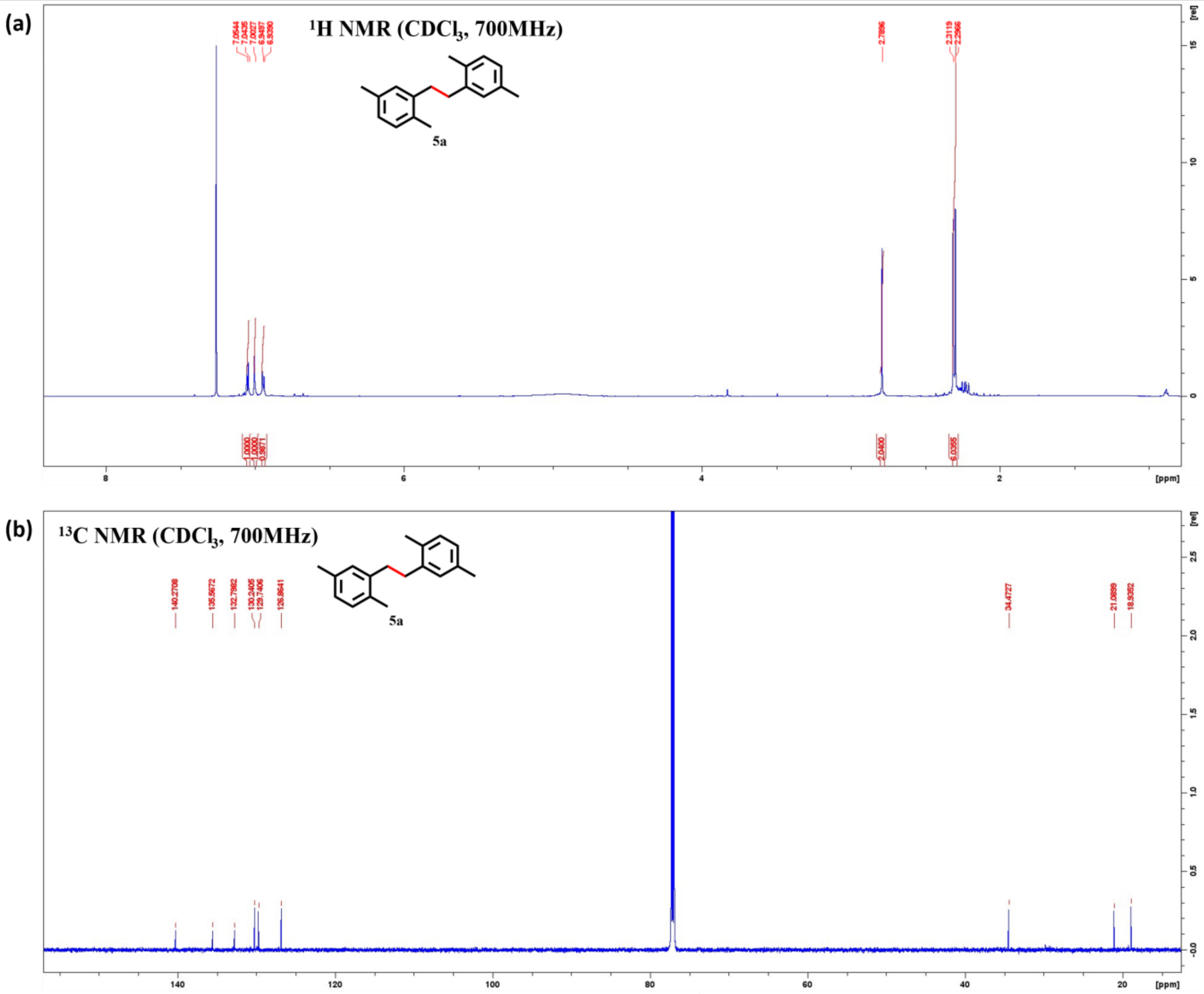

Figure S13 (a) ^{1}H NMR of 5a, and (b) ^{13}C NMR of 5a

^{1}H NMR (700 MHz, CDCl3): δ = 2.29 (s, 6H), 2.31 (s, 6H), 2.78 (s, 4H), 6.94 (s, 2H), 7.00 (s, 2H), 7.05 (s, 2H)
^{13}C NMR (700 MHz, CDCl3): δ 18.9, 21.1, 34.5, 126.8, 129.7, 130.2, 132.8, 135.5, 140.2

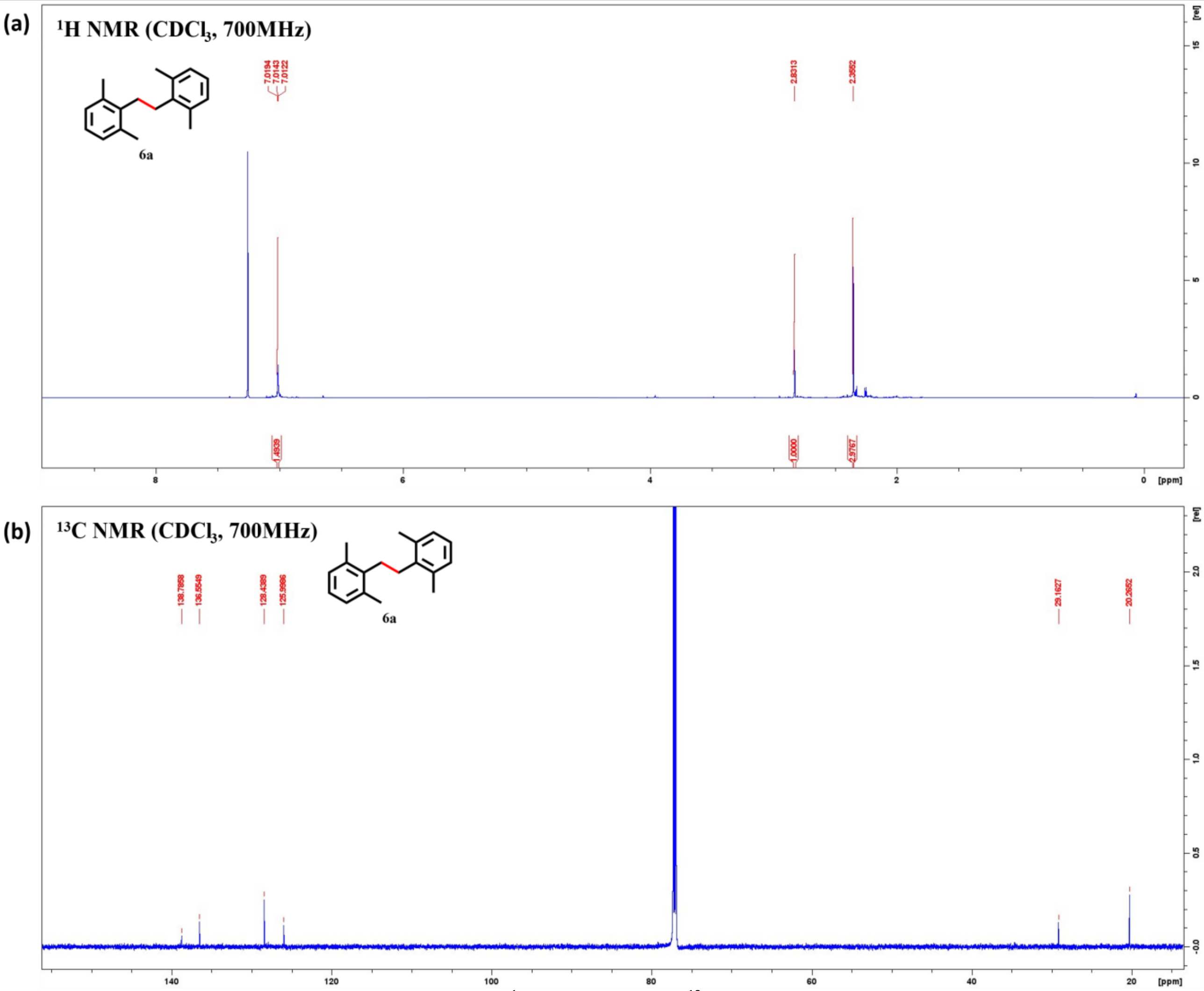

Figure S14 (a) ^{1}H NMR of 6a, and (b) ^{13}C NMR of 6a

^{1}H NMR (700 MHz, $CDCl_3$): δ = 2.35 (s, 12H), 2.83 (s, 4H), 7.01 (s, 6H)
^{13}C NMR (700 MHz, $CDCl_3$): δ 20.2, 29.2, 125.9, 128.4, 135.5, 138.7

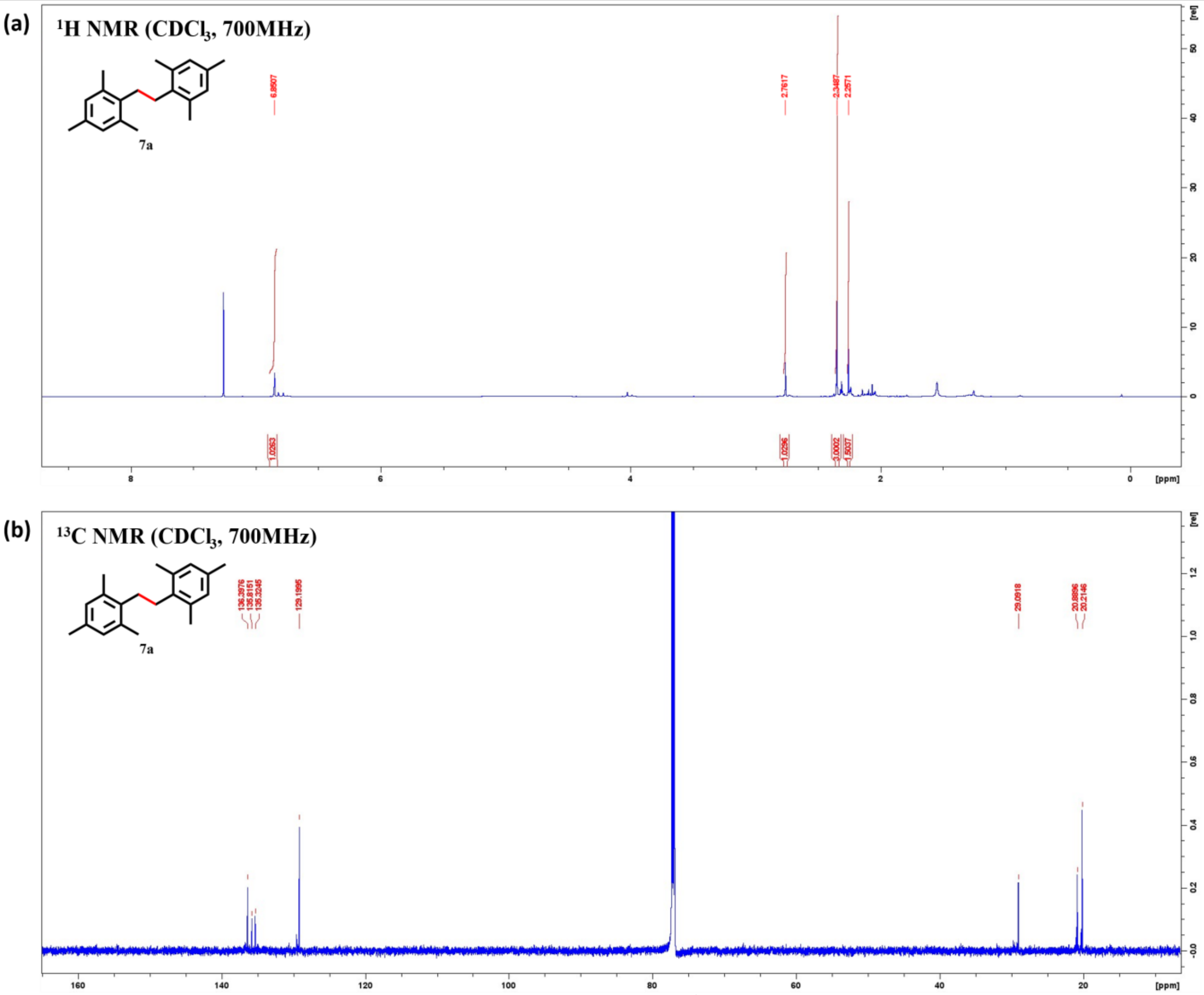

Figure S15 (a) ^{1}H NMR of 7a, and (b) ^{13}C NMR of 7a

^{1}H NMR (700 MHz, $CDCl_3$): δ = 2.25 (s, 6H), 2.35 (s, 12H), 2.76 (s, 4H), 6.85 (s, 4H)
^{13}C NMR (700 MHz, $CDCl_3$): δ 20.2, 20.9, 29.1, 129.1, 135.3, 135.8, 136.4

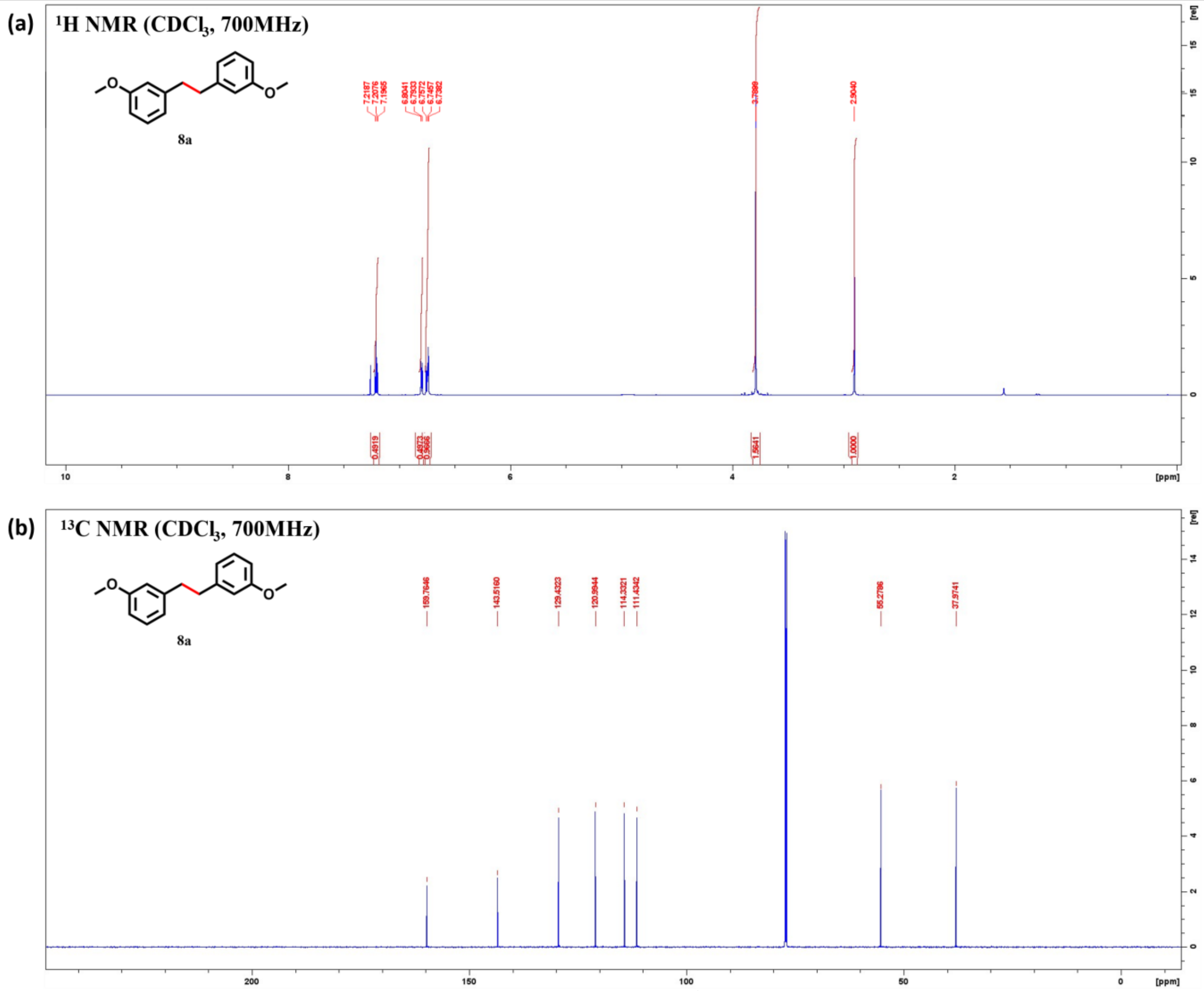

Figure S16 (a) ^{1}H NMR of 8a, and (b) ^{13}C NMR of 8a

^{1}H NMR (700 MHz, $CDCl_3$): δ = 2.90(s, 4H), 3.79 (s, 6H), 6.73-6.80 (m, 6H), 7.19-7.21 (m, 2H)
^{13}C NMR (700 MHz, $CDCl_3$) δ 37.9, 55.2, 111.4, 114.3, 120.9, 129.4, 143.5, 159.7

(a)

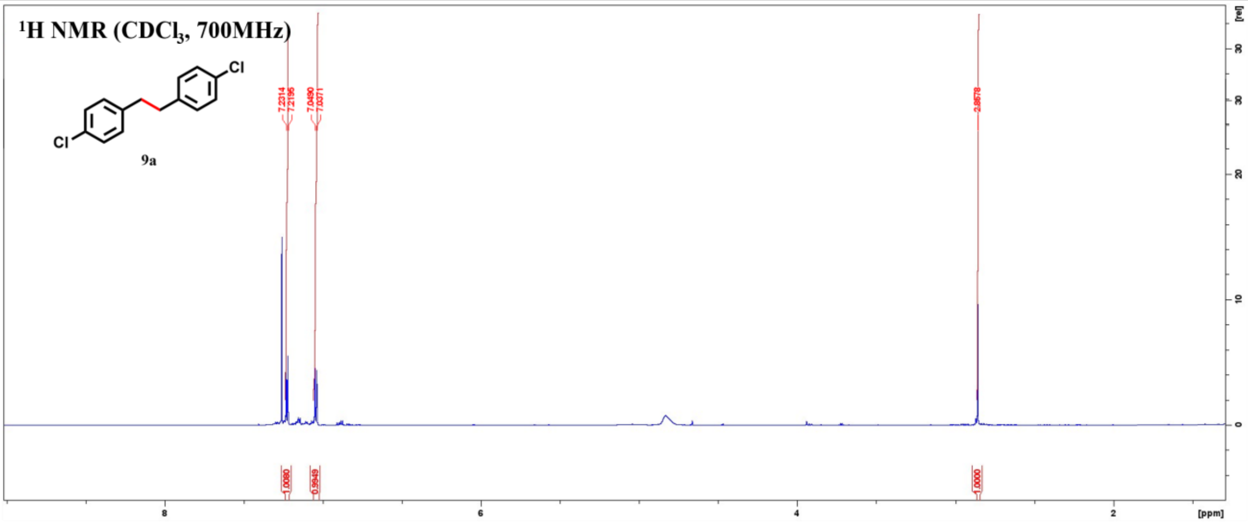

(b)

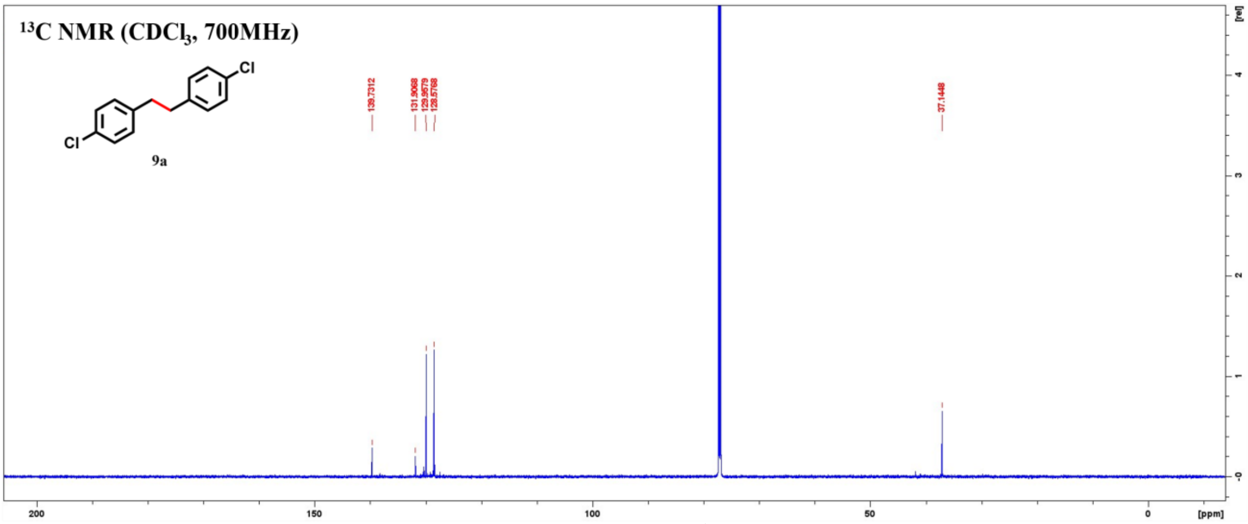

Figure S17 (a) ^{1}H NMR of 9a, and (b) ^{13}C NMR of 9a

^{1}H NMR (700 MHz, $CDCl_3$): δ = 2.85 (s, 4H), 7.03 (d, 4H), 7.21 (d, 4H)
^{13}C NMR (700 MHz, $CDCl_3$) δ 37.1, 128.5, 129.9, 131.9, 139.7

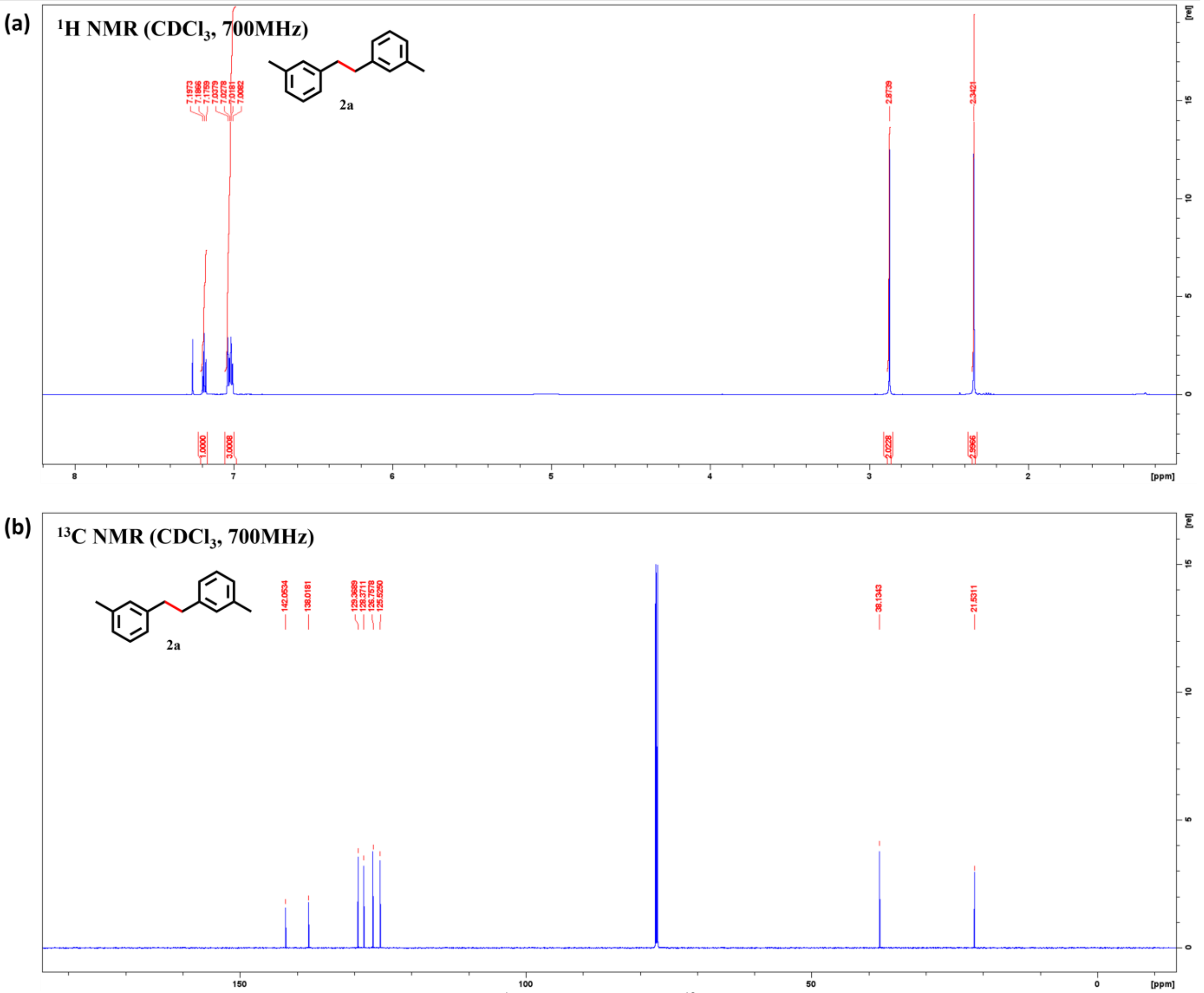

Figure S18 (a) ^{1}H NMR of 10a, and (b) ^{13}C NMR of 10a

^{1}H NMR (700 MHz, $CDCl_3$): δ = 2.98(s, 1H), 3.90 (s, 2H), 7.19 (s, 1H), 7.93 (s, 1H)
^{13}C NMR (700 MHz, $CDCl_3$) δ 37.5, 52.1, 128.2, 128.6, 129.8, 146.6, 167.1

(a)

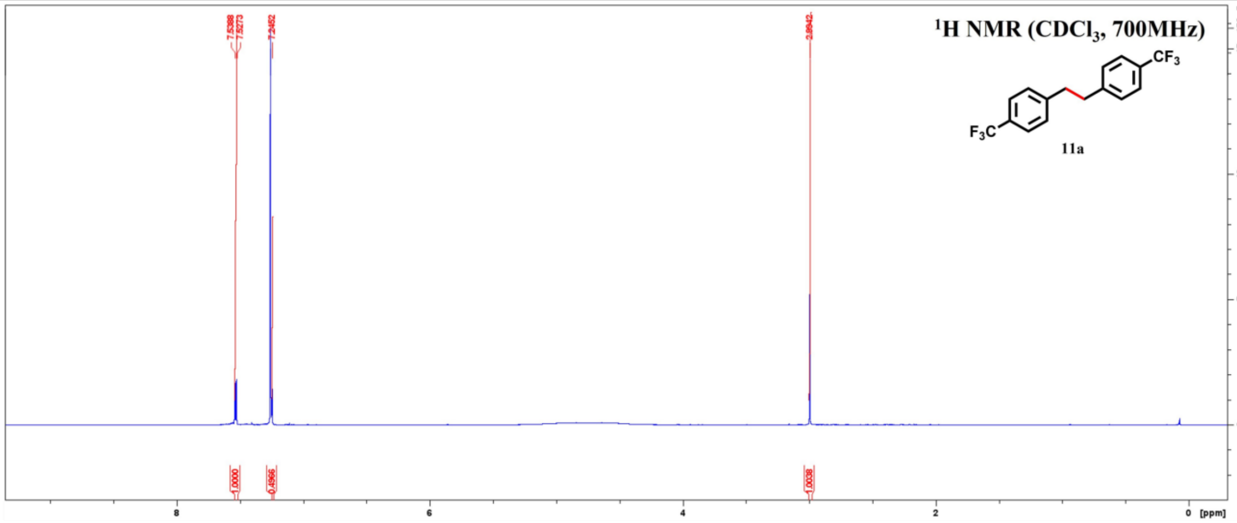

(b)

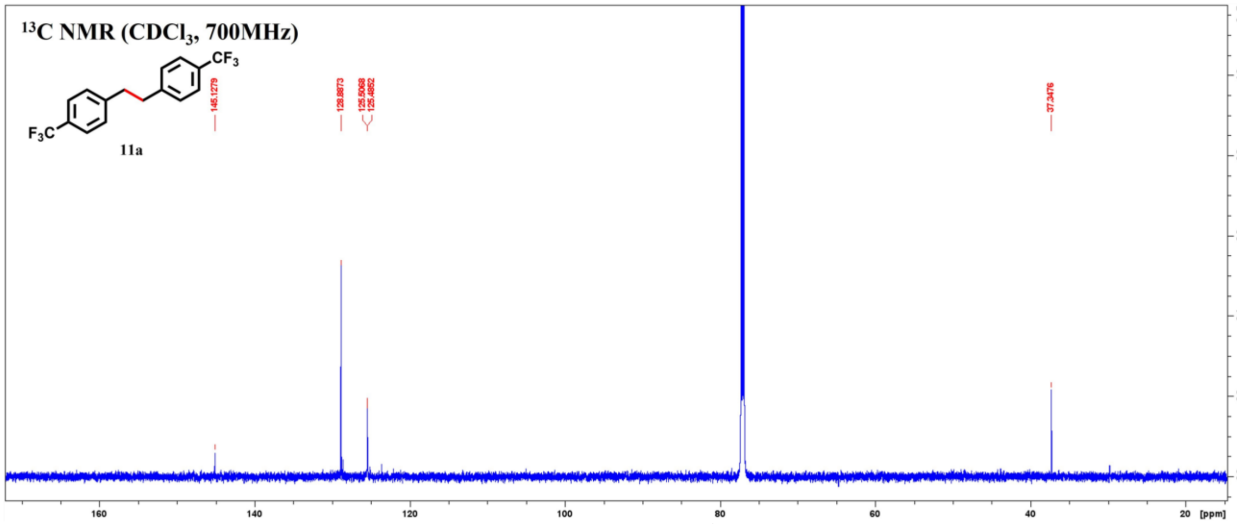

Figure S19 (a) ^1H NMR of 11a[a], and (b) ^{13}C NMR of 11a

^1H NMR (700 MHz, $CDCl_3$): δ = 2.99(s, 4H), 7.24 (d, 4H)[a], 7.53 (d, 4H)
[a]One peak overlapped with $CDCl_3$
^{13}C NMR (700 MHz, $CDCl_3$) δ 37.3, 125.4, 125.5, 128.8, 145.1

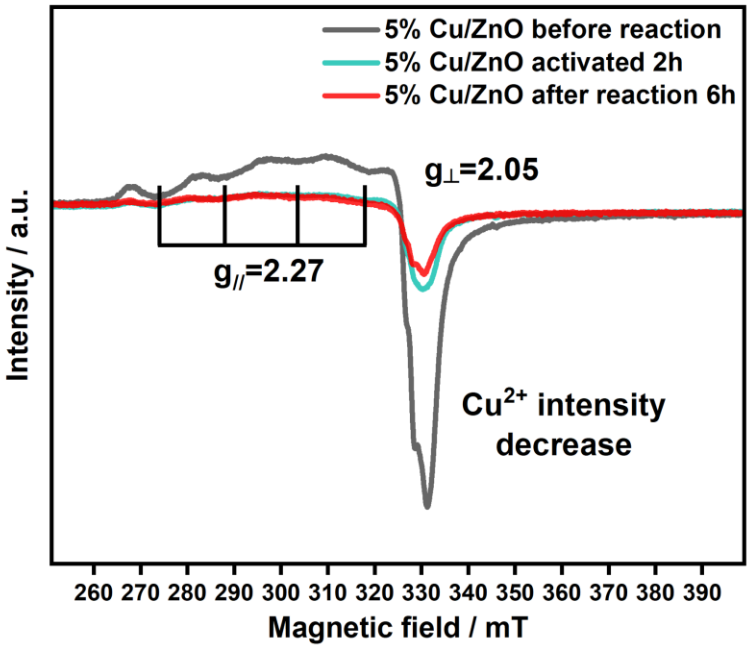

Figure S20 EPR spectra of 5% Cu/ZnO samples (before, activated and after reaction)

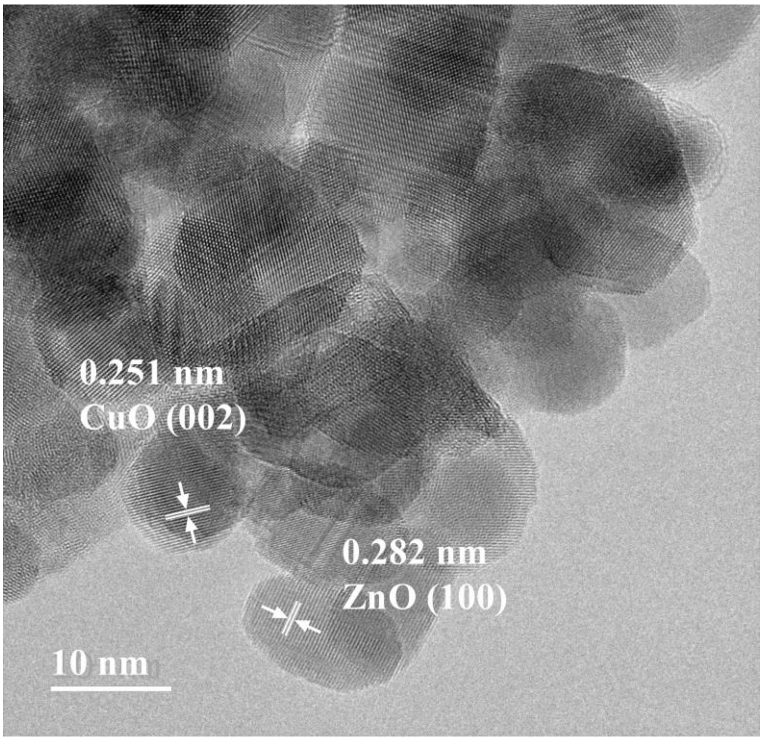

Figure S21 TEM image of Cu/ZnO after reaction sample

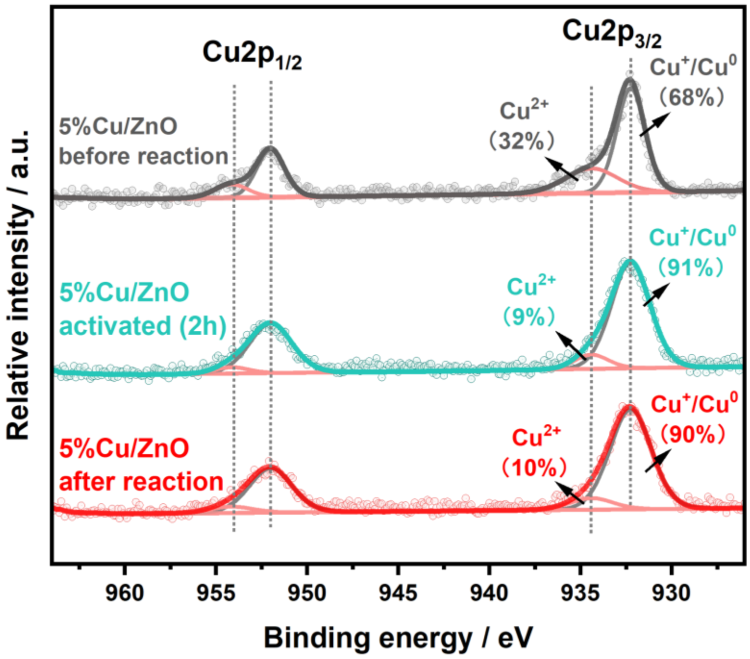

Figure S22 Deconvolution of XPS spectra in the Cu $2p_{3/2}$ region for 5% Cu/ZnO samples (before, activated and after reaction)

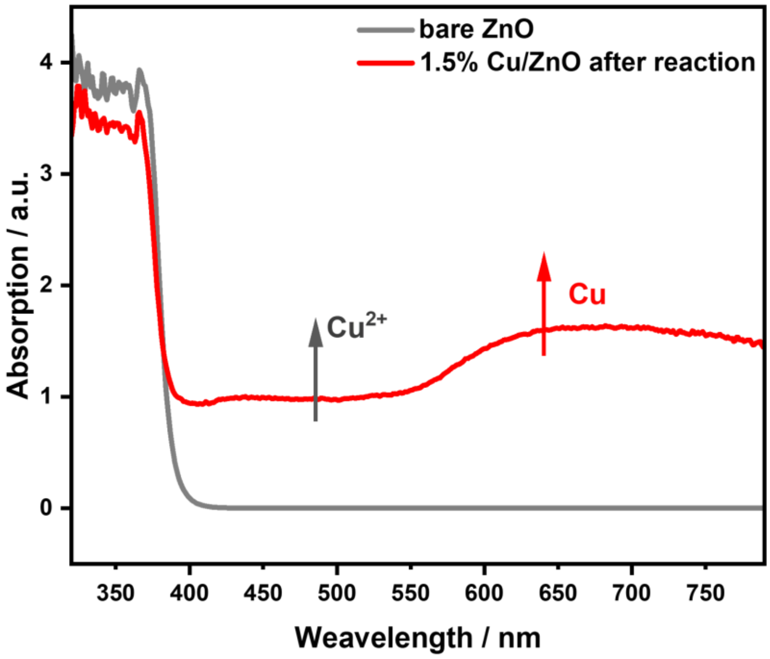

Figure S23 UV-vis spectra of bare ZnO, 1.5% Cu/ZnO after reaction sample

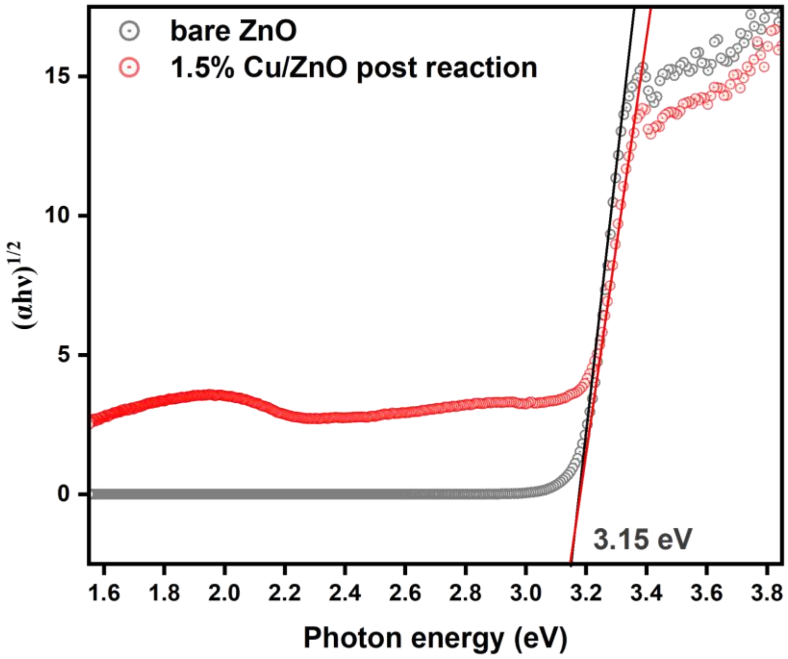

Figure S24 Kubelka-Munk function of of bare ZnO, 1.5% Cu/ZnO post reaction sample

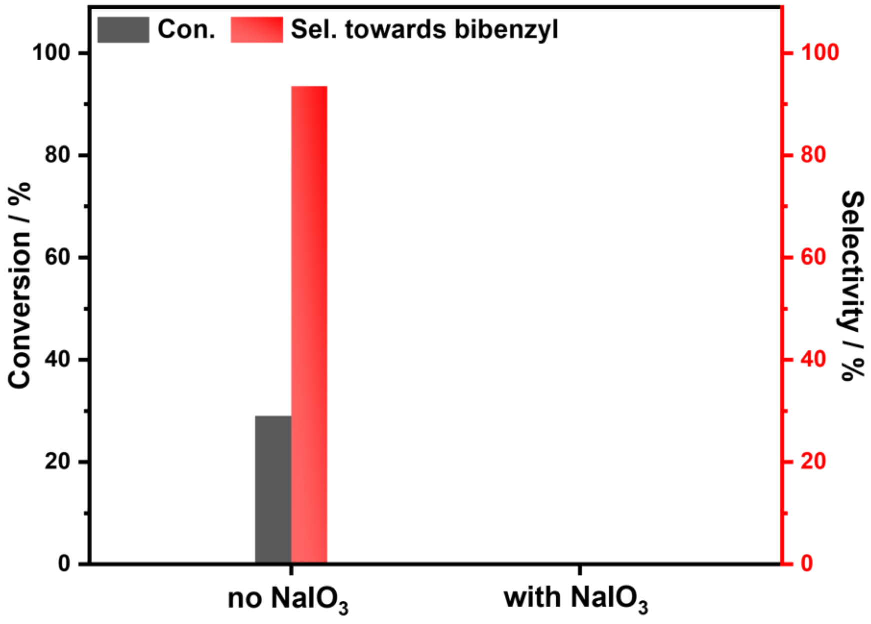

Figure S25 Electron capture experiments. Reaction conditions: 10mM reactant, with/without $NaIO_3$, 10 mg 1.5 wt% Cu/ZnO in 5 ml solvent (2-propanol: water = 1:1) under 365 nm LED irradiation for 1.5h

Table S3. Energy data of benzyl chloride and benzyl radicals on different model surface for DFT

Gas molecules	
**Molecule**	**E_{tot} (eV)**
Ph-Ch_2Cl(g)	-91.289
Ph-Ch_2(g)	-87.685

	ZnO(100)		Cu_{10}/ ZnO(100)		Cu_{10}/ TiO_2(101)	
**Adsorbate**	**E_{tot} (eV)**	**E_{ads} (eV)**	**E_{tot} (eV)**	**E_{ads} (eV)**	**E_{tot} (eV)**	**E_{ads} (eV)**
*	-425.941	-	-457.157	-	-597.048	-
Ph-CH_2Cl*	-518.511	-1.28	-549.540	-1.09	-689.342	-1.00
Ph-CH_2*	-514.813	-1.19	-547.228	-2.39	-687.105	-2.37

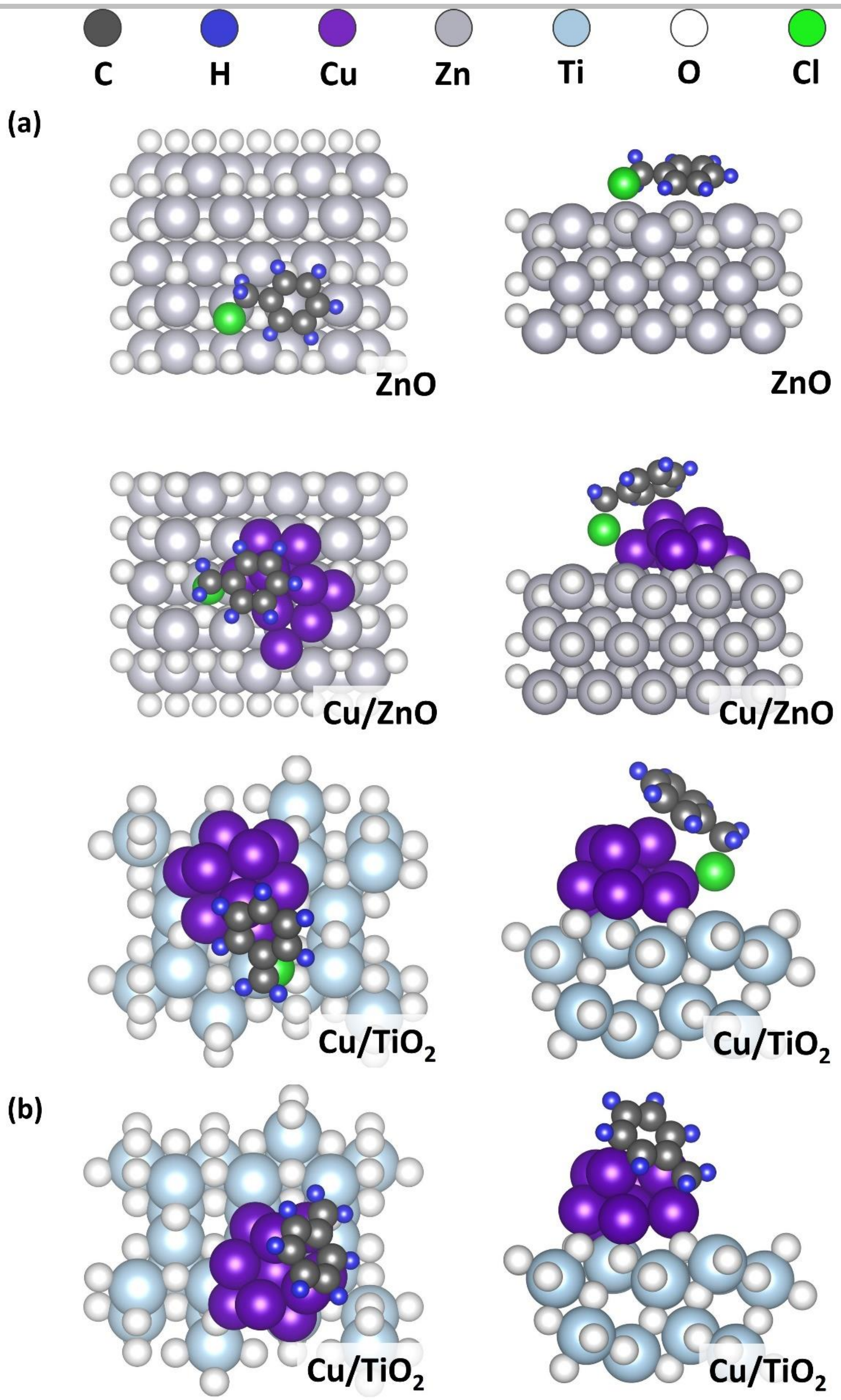

Figure S26 Optimised adsorption model of (a) benzyl chloride on selected surfaces of ZnO, Cu/ZnO and Cu/TiO_2 and (b) benzyl radical on selected surfaces of Cu/TiO_2 by DFT calculation

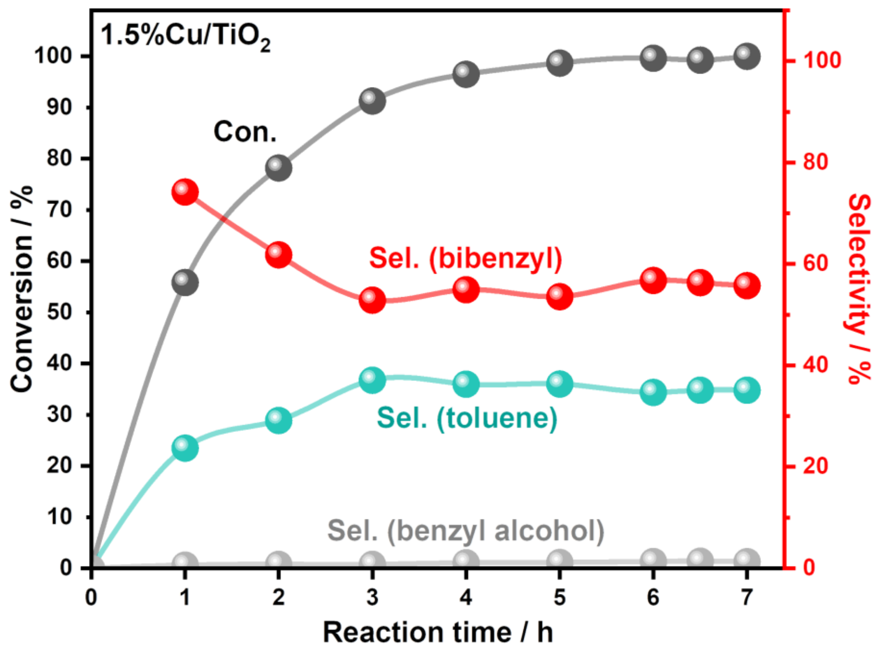

Figure S27 Temporal study of photocatalytic benzyl chloride conversion using Cu/TiO_2. Reaction conditions: 10mM reactant, 10 mg photocatalyst in 5 ml solvent (2-propanol: water = 1:1) under 365 nm LED irradiation for 7h, Ar, room temperature.

Table S4. Mulliken charge of C_7H_7Cl

Calculated system	Optimised structure[a]	Mulliken charge of C_7H_7Cl / e
C_7H_7Cl on Cu_{10} cluster	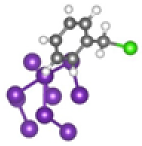	−0.086
C_7H_7Cl on Cu_{10}^- cluster	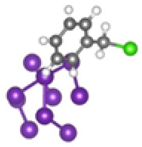	−0.111

[a] The grey, white, green, and purple spheres represent carbon atoms, hydrogen atoms, chlorine atoms and copper atoms, respectively.

References

[1] X. Chen, Y. Li, X. Pan, D. Cortie, X. Huang, Z. Yi, *Nat. Commun.* **2016**, *7*, 12273.

[2] H. Wang, H. Qi, X. Sun, S. Jia, X. Li, T. J. Miao, L. Xiong, S. Wang, X. Zhang, X. Liu, A. Wang, T. Zhang, W. Huang, J. Tang, *Nat. Mater.* **2023**, *22*, 619–626.

[3] G. Kresse, J. Furthmüller, *Comput. Mater. Sci.* **1996**, *6*, 15–50.

[4] G. Kresse, J. Furthmüller, *Phys. Rev. B* **1996**, *54*, 11169–11186.

[5] J. P. Perdew, K. Burke, M. Ernzerhof, *Phys. Rev. Lett.* **1996**, *77*, 3865–3868.

[6] G. Kresse, D. Joubert, *Phys. Rev. B* **1999**, *59*, 1758–1775.

[7] P. E. Blöchl, *Phys. Rev. B* **1994**, *50*, 17953–17979.

[8] S. L. Dudarev, G. A. Botton, S. Y. Savrasov, C. J. Humphreys, A. P. Sutton, *Phys. Rev. B* **1998**, *57*, 1505–1509.

[9] S. Grimme, J. Antony, S. Ehrlich, H. Krieg, *J. Chem. Phys.* **2010**, *132*, 154104.

[10] M. J. Frisch, G. W. Trucks, H. B. Schlegel, G. E. Scuseria, M. a. Robb, J. R. Cheeseman, G. Scalmani, V. Barone, G. a. Petersson, H. Nakatsuji, X. Li, M. Caricato, a. V. Marenich, J. Bloino, B. G. Janesko, R. Gomperts, B. Mennucci, H. P. Hratchian, J. V. Ortiz, a. F. Izmaylov, J. L. Sonnenberg, Williams, F. Ding, F. Lipparini, F. Egidi, J. Goings, B. Peng, A. Petrone, T. Henderson, D. Ranasinghe, V. G. Zakrzewski, J. Gao, N. Rega, G. Zheng, W. Liang, M. Hada, M. Ehara, K. Toyota, R. Fukuda, J. Hasegawa, M. Ishida, T. Nakajima, Y. Honda, O. Kitao, H. Nakai, T. Vreven, K. Throssell, J. a. Montgomery Jr., J. E. Peralta, F. Ogliaro, M. J. Bearpark, J. J. Heyd, E. N. Brothers, K. N. Kudin, V. N. Staroverov, T. a. Keith, R. Kobayashi, J. Normand, K. Raghavachari, a. P. Rendell, J. C. Burant, S. S. Iyengar, J. Tomasi, M. Cossi, J. M. Millam, M. Klene, C. Adamo, R. Cammi, J. W. Ochterski, R. L. Martin, K. Morokuma, O. Farkas, J. B. Foresman, D. J. Fox, **2016**, Gaussian 16, Revision C.01, Gaussian, Inc., Wallin.

[11] K. Burke, *J. Chem. Phys.* **2012**, *136*, 150901.
